# Impact of converging sociocultural and substance-related trends on US autism rates: combined geospatiotemporal and causal inferential analysis

**DOI:** 10.1007/s00406-022-01446-0

**Published:** 2022-07-02

**Authors:** Albert Stuart Reece, Gary Kenneth Hulse

**Affiliations:** 1 Division of Psychiatry, University of Western Australia, Crawley, WA 6009 Australia; 2 School of Medical and Health Sciences, Edith Cowan University, Joondalup, WA 6027 Australia

**Keywords:** Cannabis, Cannabinoid, Δ9-tetrahydrocannabinol, Cannabigerol, Pathways and mechanisms

## Abstract

Whilst cannabis is known to be toxic to brain development, it is unknown if it is driving rising US autism rates (ASMR). A longitudinal epidemiological study was conducted using national autism census data from the US Department of Education Individuals with Disabilities Act (IDEA) 1991–2011 and nationally representative drug exposure (cigarettes, alcohol, analgesic, and cocaine abuse, and cannabis use monthly, daily, and in pregnancy) datasets from National Survey of Drug Use and Health and US Census (income and ethnicity) and CDC Wonder population and birth data. Analysis was conducted in R. 266,950 were autistic of a population of 40,119,464 8-year-olds in 1994–2011. At national level after adjustment, daily cannabis use was significantly related to ASMR (*β* estimate = 4.37 (95%C.I. 4.06, 4.68), *P* < 2.2 × 10^–16^) as was first pregnancy trimester cannabis exposure (*β* estimate = 0.12 (0.08, 0.16), *P* = 1.7 × 10^–12^). At state level following adjustment for cannabis, cannabigerol (from *β* estimate = – 13.77 (– 19.41, 8.13), *P* = 1.8 × 10^–6^) and Δ9-tetrahydrocannabinol (from *β* estimate = 1.96 (0.88–3.04), *P* = 4 × 10^–4^) were significant. Geospatial state-level modelling showed exponential relationship between ASMR and Δ9-tetrahydrocannabinol and cannabigerol exposure. Exponential coefficients for the relationship between modelled ASMR and Δ9-tetrahydrocannabinol and cannabigerol exposure were 7.053 (6.39–7.71) and 185.334 (167.88–202.79; both *P* < 2.0 × 10^–7^). E*-*values are an instrument related to the evidence for causality in observational studies. High E*-*values were noted. Dichotomized legal status was linked with elevated ASMR. Data show cannabis use is associated with ASMR, is powerful enough to affect overall trends, and persists after controlling for other major covariates. Cannabinoids are exponentially associated with ASMR. The cannabis–autism relationship satisfies criteria of causal inference.

**Supplementary Information:**

The online version contains supplementary material available at 10.1007/s00406-022-01446-0.

## Introduction

It is well known that the incidence of autistic spectrum disorder is increasing in the USA, with current annual rates as high as 1.68% being reported nationwide by Centers for Disease Control, Atlanta, Georgia (CDC) [1]. Indeed up to 4.5% of 8-year-old boys in New Jersey have been diagnosed with this disorder [1]. For reasons which are unclear, the syndrome is more common in boys than girls perhaps related to the many extra neurological genes on the X-chromosome, which is randomly inactivated in females thereby providing a wider range of spare alleles from which to support neurological development [2].

Whilst the literature identifies several causes which contribute to the incidence of autism, including obesity, maternal diabetes, advanced parental age, twin linkage, bleeding, having another autistic sibling, higher income, and exposure to some drugs including cannabinoids [3–7], the primary drivers of the present surge have remained largely elusive.

Of concern, all three longitudinal studies of brain development following prenatal cannabis exposure (PCE) have identified adverse neurological outcomes mimicking attention deficit hyperactivity disorder (ADHD) and autistic spectrum features [8]. At a time of major commercialization of the cannabis industry, such findings must be of particular concern.

It is of interest that a recent population-wide study of all births in Ontario 2007–2012 using coarsened exact matching and controlling for a wide variety of socioeconomic, medical, maternal age, maternal psychiatric, other substance use, and obstetric covariates found a 51% higher adjusted rate of autistic spectrum disorders (adjusted hazard ratio = 1.51 (95% CI 0.17–1.96)) following cannabis-exposed pregnancies which was invariant across all socioeconomic strata [9].

Because these syndromes are not usually identified prior to the age of 8 years, there is inevitably a lengthy delay in reporting the current state of the epidemic.

At the time of conducting our analysis, we were aware that drug exposure was highly correlated to ethnocultural factors and that PCE was known to be rising across USA. It was felt to be important to take such considerations into account in conducting our analysis.

Our primary hypothesis was that increasing substance and/or cannabinoid exposure might constitute a primary underlying driver of US autism rate (ASMR) across time. This hypothesis was formulated prior to data analysis. We wished to explore the effects and relative contribution of external demographic and socioeconomic covariates in a formal geotemporospatial framework.

## Methods

### Data sources

State autism rates were derived from the US Department of Education Individuals with Disabilities (IDEA) database [10]. State population data from the US Census Bureau were used to calculate national rates. State population, ethnicity and median household income data was sourced from US Census via the tidycensus package in “R” from Comprehensive “R” Archive Network (CRAN). Data on national age of child-bearing was sourced from the births registries of the CDC Wonder website [11]. Drug use data in various demographic subgroups and in pregnancy was taken from the nationally representative National Survey of Drugs and Health (NSDUH) conducted each year by the Substance Abuse and Mental Health Services Administration (SAMHSA) and particularly from the online interactive Substance Abuse and Mental Health Data Archive (SAMHDA [12]). Data on national cannabinoid concentrations was from Drug Enforcement Agency [13, 14]. Missing data were casewise deleted in linear (lm) and panel (plm) regression except where otherwise described.

State cannabinoid exposure estimates were derived by multiplying the monthly cannabis use rate by state by the concentration of the various cannabinoids obtained in Federal seizures. Data on Δ9-tetrahydrocannabinol (Δ9THC), cannabinol (CBN), cannabidiol (CBD), cannabigerol (CBG), cannabichromene (CBC) and tetrahydrocannabidivarin (THCV) were available [13, 14].

Ethnicity was defined by SAMHSA and US Census. These official definitions of ethnicity were used in analysis.

### Statistics

This study was conducted in 2019. Data was processed using “R Studio” version 1.2.5042 based on “R” version 4.0.0 [15]. All graphs were prepared in ggplot2 package [16] from the tidyverse [17] and 3-D graphs were drawn in NCSS software [18]. All graphs and tables are original and have not been previously published elsewhere. Variables were log transformed as guided by the Shapiro test. Details of R-packages used are provided in the online statistical methods. Mixed effects models were performed using R package nlme using State as a grouping variable weighted by inverse probability weights as described below [19]. Two-step panel regression was conducted for space–time panel data using package plm [20, 21]. For panel regression the pooling model was used, effect was over both space and time, random method was that of Swarmy and the instrumental method was that of Amemiya. These settings are required by the software or were found on preliminary analyses to give optimal output precision. Geospatial links were constructed canonically using the poly2nb function from spdep [22]. Spatial links were edited with Alaska and Hawaii elided (moved) conceptually to Oregon and Washington and to California, respectively, both to reflect sociocultural relationships and to prevent areal zones with no spatial relationships which complicates geospatial analysis. Generalized two-step geospatial regression was performed using the spreml function from package splm [23, 24], including both spatial autocorrelation errors and spatial lags and random effects using the error structure of Kapoor, Kelejian and Prucha and with the method of Baltagi, Pfaffermayr, Jong and Song with initial values of zeros (sem2srre) [25]. Model specification was checked with Lagrange multiplier tests and models were compared by their log-likelihood (logLik) ratios at model optimization using the spatial Hausman test (sphtest). Model reduction was by the classical technique with sequential deletion of the least significant term.

Two-step regression is a powerful well-established technique which utilizes instrumental variables that are thought to more accurately reflect the real situation underlying the listed covariates. It has been used in panel and geospatial models in this report due to overwhelming evidence (presented below) of very different cannabis use patterns by ethnicity to more accurately explore the underlying drug exposure relationships.

Predicted fitted values from final models were calculated by matrix multiplication inserting appropriate values alongside matching model terms.

### Causal inference

Inverse probability weighting (IPW) was conducted using the R package ipw [26]. IPW values were calculated using the last month cannabis use as the exposure of interest in a time-dependent manner. The numerator was a series of additive terms including four drug variables excluding cannabis exposure, four ethnicities, median household income and five ethnic cannabis exposure terms. The denominator included this list together with monthly cannabis exposure. Interactive models included a four-way interaction between tobacco, alcohol, cannabis and analgesic consumption. Weight truncation was not required. All mixed effects and robust models were inverse probability weighted. Robust generalized linear regression was performed in the survey package (using svyglm) with State as the grouping variable utilizing the IPW weights [27].

EValue determination was performed using the R package EValue [28–30]. As eValue estimation of regression coefficients requires a model standardized deviation, this could not be performed on svyglm models; it was performed instead on mixed effects models structured and weighted similarly to the svyglm models.

*P* < 0.05 was considered significant.

## Results

### Input data

The national rate of autistic spectrum disorder was derived from the IDEA database combined with state population data obtained from US Census and used to compute national rates of autism. It was combined with other data as shown in eTable 1 and graphed in eFigure 1. The IDEA dataset for the 50 US states was almost complete for the 18 years 1994–2011. Only five data points were missing for this period: New Hampshire in 1994, Montana 2006, Vermont in 2007 and 2008, and Wyoming 2010 and these were filled by temporal kriging (mean substitution). This dataset comprehended 266,950 autistic children of a total US population of 40,119,464 8-year-olds, a mean rate of 66.5/10,000, for the period 1994–2011.

Since the IDEA database began in 1991 and terminated in 2011, it was extended through to 2018 using conservative published national projections [31] which are actually below the most recent CDC estimate (1.31% in 2014 v. 1.68% in [1]). Data on cannabis use by ethnic group, daily cannabis smoking and cannabis use in pregnancy was only available from SAMHSA at the national level, which indicated that these variables needed to be analysed at the national level. Authoritative and nationally representative surveys have shown repeatedly that rates of cannabis use in pregnancy closely parallel those in the general community [32–38].

**Fig. 1:**
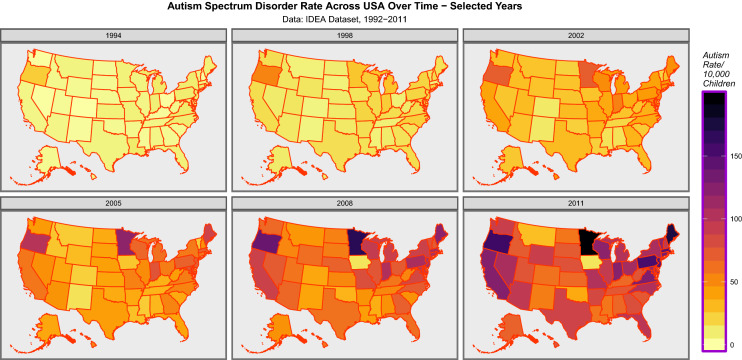
Map sequence of autism rates across USA selected years 1992–2011. Data from IDEA Dataset in reference 4

Figure 1 presents a sequential map series showing the progress of autism across USA 1992–2011.

**Fig. 2:**
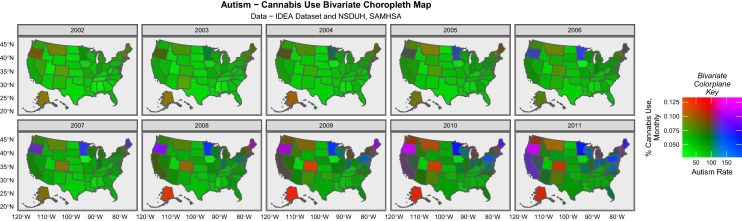
Bivariate choropleth maps of the relationship between autism and cannabis use over time

Figure 2 presents a bivariate map series of the autism rate together with the cannabis use rate and one notes that both are elevated in the northeast and northwest of the country (pink and purple areas).

**Fig. 3:**
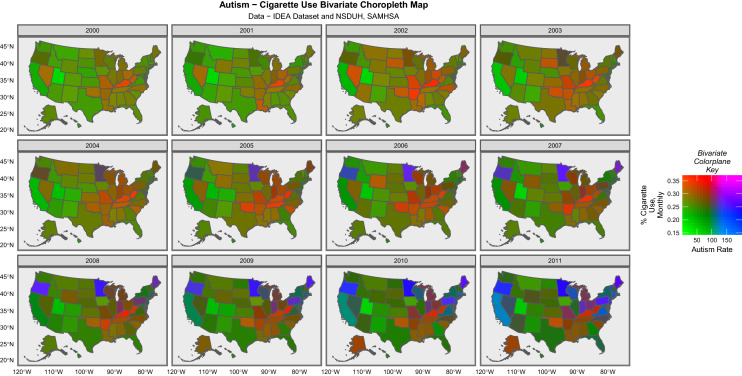
Bivariate choropleth maps of the relationship between autism and cigarette use over time

Figure 3 presents a similar bivariate map of USA showing autism and cigarette use plotted together. As cigarette use declines, this map appears to be “turning bluer” than the previous map.

**Fig. 4:**
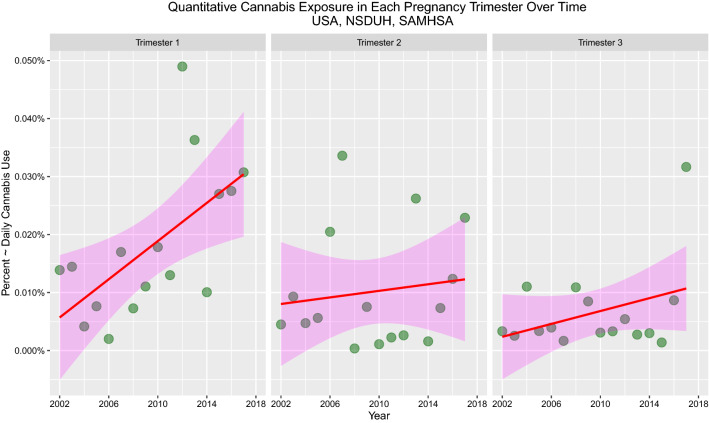
Plots of cannabis use in each pregnancy trimester over time. Data from SAMHDA from SAMHSA

The United Nations 2019 World Drug Report clearly demonstrates that recent American use of cannabis relates primarily to increased daily use [39]. SAMHSA provide data that stratify the monthly frequency of cannabis use into groups as non-user, 1–2 days, 3–5 days, 6–19 days and 20–30 days shown in eFigure 2. The confidence intervals are taken directly from SAMHDA. Again, one notes that Asian-Americans smoke less cannabis 20–30 days per month and more are non-users. Using the midpoint of these daily intervals as a multiplicand, it is possible to calculate the mean daily use of each ethnic group over time with the results shown in Fig. 4 and eFigures 2 and 3. Clear differences in mean daily cannabis use by ethnicity are evident.

As disclosed by United Nations Office of Drugs and Crime (UNODP), the pattern of cannabis use matters. SAMHDA data show that in 2017 about 92.6% of Americans smoked cannabis to a trivial extent (≤ 3 days/month) and 7.35% smoked ≥ 3 days/month (eTable 2).

These data allow the calculation of an Ethnic Cannabis Exposure Score which can be plotted against a State–Time index and against time (eFigure 4A and 4B). These data show that without exception in each state, the Ethnic Cannabis Exposure Score rose across time. The red line in the centre of Panel B shows the median trajectory as a loess curve of best fit.

### Regression results

Linear regression was used to investigate the association between daily cannabis use and ethnicity. The covariates were time and ethnicity. eTable 3 shows the results in a model quadratic in time and confirms highly significant differences in cannabis use by ethnicity (from *β* estimate = 1.67 (95% CI 1.45–1.89), *P* < 2.2 × 10^–16^; quadratic superior to linear model, ANOVA *F* = 2.147, df = 13, *P* = 0.019).

eFigure 5 shows that high intensity cannabis use is falling amongst teenagers, but rising in older age groups. eFigure 6 confirms these age-dependent trends in the first trimester of pregnancy which shows more cannabis use than later trimesters. eFigure 7 has been drawn from CDC birth data and confirms the trend of childbirth to be occurring at older maternal ages. In the light of the findings of eFigure 5, this implies that these women are moving up into a higher cannabis use age bracket.

**Fig. 5:**
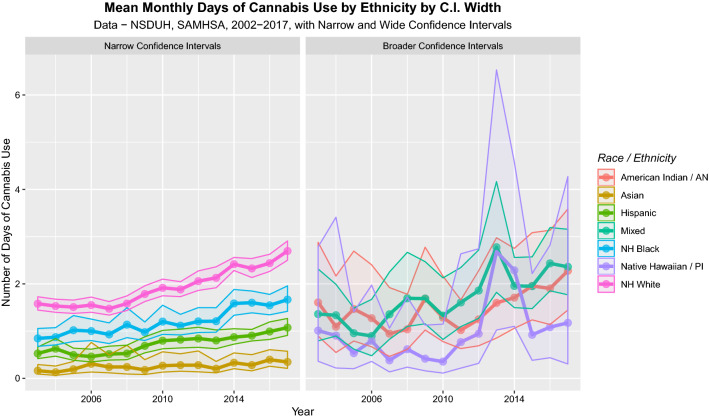
Mean cannabis use by ethnicity. Data from SAMHDA from SAMHSA

Figure 5 presents the mean data for cannabis use by pregnancy trimester for all age groups and confirms that first trimester cannabis use is rising with time, a trend not seen at later trimesters. The SAMHSA data for 2015 is incomplete, so this point has been filled by mean substitution (0.027). The correlation between time and the rising use of cannabis in pregnancy is *R* = 0.6115 (*P* = 0.001). The slope of the first trimester regression line is significantly different to that in the third trimester (*β* estimate = – 4.97 × 10^–8^ (– 8.44E-08 to – 1.5E-08), *P* = 0.007, model Adj. *R*^2^ = 0.174, *F* = 4.31, df = 3.44, *P* = 0.009).

These data invite exploration by regression analysis. Panel regression was utilized as time is an implicit variable rather than an explicit one (important in small data tables), and one can easily include both temporal lags and instrumental variables in the R package plm. Only a limited number of variables can be included because of the small number of observations. The Ethnic Cannabis Exposure Score was multiplied by the THC Potency to capture the effect of rising THC concentrations. The variable was called the “Ethnic Cannabis Score THC Potency”. Cigarettes, the cannabis index, analgesics, three races and median household income have been included as covariates for 1994–2018. When the regression is performed for the national autism rate in this manner the results indicated in Table 1 are obtained. A very high level of statistical significance of all the variables is noted (all *P* < 2.2 × 10^–16^).

**Table 1:** National panel regression model results

Instrumental ± lagged variables	Parameter	Parameter		
		estimate	CI	*P* value
General population model				
2 lags, 1 interaction				
Lag (Cannabis_Monthly), 0:2	Cigarettes_Monthly	31.83	(29.79–33.87)	< 2.2e-16
Lag (Δ9THC_Exposure), 0:2	African-American_Ethnicity	11.15	(10.6–11.7)	< 2.2e-16
Lag (Cannabigerol_Exposure), 0:2	Ethnic_Cannabis_Score_THC_Potency	4.37	(4.06–4.68)	< 2.2e-16
Cocaine_Annual	Hispanic_Ethnicity	0.83	(0.77–0.89)	< 2.2e-16
	Median_Household_Income	1.5E-05	(1.4E-05–1.6E-05)	< 2.2e-16
	Non-Medical_Use_of_Analgesics	– 2.98	(– 3.3–2.7)	< 2.2e-16
	Caucasian-American_Ethnicity	– 14.79	(– 15.3–14.3)	< 2.2e-16
	Cigarettes_Monthly: Ethnic_Cannabis_Score_THC_Potency	– 18.65	(– 19.9–17.4)	< 2.2e-16
First trimester pregnancy exposure				
2 lags, 1 interaction				
Lag(First_Trimester_Cannabis_Exposure), 0:2	First_Trimester_Cannabis_Exposure: THC_Potency	– 0.06	(– 0.08–0.04)	< 2.2e-16
Lag(THC_Potency), 0:2	Caucasian-American_Ethnicity	– 6.19	(– 7.07–5.31)	< 2.2e-16
Lag(White_Ethnicity), 0:2	First_Trimester_Cannabis_Exposure	0.12	(0.08–0.16)	1.7E-12
Lag(Hispanic_Ethnicity), 0:2	Cocaine_Annual	0.25	(0.15–0.35)	3.9E-08

*0:2* represents 0–2 years temporal lag, *THC* tetrahydrocannabinol, *Δ9THC* Δ9- tetrahydrocannabinol, *CI* 95% confidence interval

Panel regression may also be used to model the relationship between ASMR and first trimester cannabis use. The covariates in this model were first trimester cannabis use, THC potency, median household income, cocaine and analgesic use, and the three most common races (Caucasian-American, African-American and Hispanic-American). This model has one interaction between first trimester cannabis use and THC potency and 2 years of lag. The instrumental variables along with the highly significant results are listed in Table 1.

### Robustness analysis

A robustness analysis on these data using published high and low estimates of the national autism rate for 1994–2018 derived from projections from states where cannabis was illegal and those where it was legal, respectively [31], confirmed these conclusions (eTable 4).

### Geospatial regression

Naturally, we were interested to explore if these relationships extended to an analysis at state level. eFigure 8 sets out the geospatial links and weights used.

Geospatial regression was performed in 2002–2011 with results shown in eTable 5 using five drugs—cigarettes, alcohol abuse, monthly cannabis, misuse of analgesics, cocaine—and the five races—Caucasian-American, African-American, Hispanic-American, Asian-American and American Indians and Alaskan Natives—and median household income were considered as covariates, and instrumental variables were used for monthly cannabis use, Δ9THC and cannabigerol and the annual Ethnic Cannabis Exposure Score was used to control for cannabis exposure arising in relation to ethnic origin. A three-way interaction term included cigarettes, cannabis and opioids. As shown in eTable 5, significant results for cannabis were obtained (from *β* estimate = 8.41 (3.08–13.74), *P* = 0.002) at 2 years lag.

Clearly in such a study, one is concerned that ethnocultural factors relating to increased drug exposure in certain communities might be acting in addition to ethnopharmacogenomic factors relating to different responses to, or processing of, addictive drugs. To control at least in part for this effect, we performed a further regression not with the states’ racial composition, but with the Ethnic Cannabis Exposure Score described above. The instrumental variable list was similar to that described above. These results are shown in eTable 6, where terms including cannabis are noted to be significant (from *β* estimate = 10.88 (5.97–15.79), *P* = 1.4 × 10^–5^) at 2 years lag, cannabis is independently significant alone (*β* estimate = 0.63 (0.13–1.13), *P* = 0.014) and the Ethnic Cannabis Exposure Score is highly significant at all lags (from *β* estimate = 0.17 (0.09–0.26), *P* = 4.6 × 10^–5^).

Finally, we were interested to learn if the inclusion of specific cannabinoids in the model would be significant when race and median household income were included. Geospatial links were derived from the R spdep package and edited as shown in eFigure 8A to achieve the final spatial links shown in eFigure 8B. The regression results from spatial two-stage and lagged models are shown in Table 2 with full model details provided in eTable 7. Instrumental variables included individual terms for ethnic cannabis exposure and are indicated in the table. Terms including cannabinoids are significant in an unlagged model (from *β* estimate = – 13.77 (– 19.41 to – 8.13), *P* = 1.8 × 10^–6^) and across all models Δ9THC and cannabigerol are independently significant (from *β* estimate = 1.96 (0.88–3.04), *P* = 4 × 10^–4^ and β estimate = 0.81(0.34–1.28), *P* = 9 × 10^–4^). Spatial Hausman tests confirm that the unlagged model is superior to models lagged to 2 and 4 years (ChiSq. = 66.879, df = 9, *P* = 3.21 × 10^–11^ and ChiSq. = 626.46, df = 9, *P* = 8.744 × 10^–129^).

**Table 2:** Geospatial state-based regression of autism rate by individual cannabinoids, race and income

General	Parameters			
Instumental ± lagged variables	Parameter	Estimate	95% CI	*P* value
0 lags				
Cannabis, monthly	NHAsian Ethnicity	0.43	(0.33–0.53)	< 2.2e-16
Δ9THC	NHWhite Ethnicity	2.01	(1.42–2.6)	1.5E-11
Cannabigerol	Cannabigerol: Alcohol_Abuse	– 13.77	( – 19.41 to – 8.13)	1.8E-06
NHWhite_Score	Alcohol_Abuse	– 44.35	( – 65.89 to – 22.81)	5.5E-05
NHBlack_Score	Cannabigerol	0.81	(0.34–1.28)	9.0E-04
Hispanic_Score	NHAIAN Ethnicity	– 0.04	( – 0.06 to – 0.02)	0.002
NHAsian_Score	cigmon: Cannabigerol: Alcohol_Abuse	8.91	(2.79–15.03)	0.004
NHAIAN_Score	Δ9THC	4.59	(1.41–7.77)	0.005
	Cigarettes: Δ9THC	– 16.23	( – 28.64 to – 3.82)	0.010
	Δ9THC: Cannabigerol	0.94	(0.21–1.67)	0.011
	Cigarettes: Δ9THC: Cannabigerol	– 3.39	( – 6.21 to – 0.57)	0.018
2 lags				
cannabis, monthly, 0:2	NHAsian Ethnicity	0.42	(0.3–0.54)	3.1E-12
Δ9THC, 0:2	NHWhite Ethnicity	1.95	(1.22–2.68)	1.2E-07
Cannabigerol, 0:2	Alcohol_Abuse	– 43.92	( – 69.97 to – 17.87)	0.001
NHWhite_Score, 0:2	NHAIAN Ethnicity	– 0.06	( – 0.1 to – 0.02)	0.001
NHBlack_Score, 0:2	Cannabigerol: Alcohol_Abuse	– 11.24	( – 18.12 to – 4.36)	0.001
Hispanic_Score, 0:2	Δ9THC	1.14	(0.36–1.92)	0.005
NHAsian_Score, 0:2	Cannabigerol	0.81	(0.22–1.4)	0.007
NHAIAN_Score, 0:2	Δ9THC: Cannabigerol	0.25	(0.03–0.47)	0.023
	NHAfrican-American Ethnicity	0.08	(0–0.16)	0.046
4 lags				
cannabis, monthly, 0:4	NHAIAN Ethnicity	– 0.11	( – 0.13 to – 0.09)	9.0E-15
Δ9THC, 0:4	NHAsian Ethnicity	0.37	(0.23–0.51)	1.9E-07
Cannabigerol, 0:4	NHWhite Ethnicity	1.52	(0.74–2.3)	1.0E-04
NHWhite_Score, 0:4	Cannabigerol: Alcohol_Abuse	– 22.68	( – 34.89 to – 10.47)	3.0E-04
NHBlack_Score, 0:4	Δ9THC	1.96	(0.88–3.04)	4.0E-04
Hispanic_Score, 0:4	Alcohol_Abuse	– 72.45	( – 114.28 to – 30.62)	7.0E-04
NHAsian_Score, 0:4	Cigarettes: Cannabigerol: Alcohol_Abuse	71.65	(25.41–117.89)	0.002
NHAIAN_Score, 0:4	Cigarettes: Δ9THC	– 6.44	( – 10.63 to – 2.25)	0.003
	Cigarettes: Alcohol_Abuse	214.56	(56.98–372.14)	0.008
0 lags, 0 instrumental variables				
	NHAIAN	– 0.14	( – 0.17 to – 0.1)	2.9E-14
	Alcohol_Abuse	– 53.52	( – 68.57 to – 38.47)	3.2E-12
	CBG: Alcohol_Abuse	– 13.87	( – 17.85 to – 9.89)	8.5E-12
	Asian.Am.Cannabis	2.60	(1.79–3.42)	4.3E-10
	Cauc.Am.Cannabis	– 3.23	( – 4.27 to – 2.19)	1.1E-09
	Hispanic.Am.Cannabis	2.96	(1.99–3.93)	2.2E-09
	NHAsian	0.34	(0.22–0.45)	5.6E-09
	AIAN.Am.Cannabis	0.48	(0.32–0.65)	7.1E-09
	Δ9THC	2.08	(1.23–2.92)	1.4E-06
	Afric.Am.Cannabis	0.30	(0.15–0.45)	8.8E-05
	NHWhite	1.25	(0.55–1.94)	0.0004
	Δ9THC:Cannabigerol	0.24	(0.06–0.41)	0.0098

*NH* non-Hispanic, *Am* American, *NHAIAN* non-Hispanic-American Indian/Alaskan-Native, *0:2* 0–2 years temporal lag, *Δ9THC* Δ9-tetrahydrocannabinol, *CI* 95% confidence interval, *0:4* 0–4 years temporal lag

It was also of interest to consider the outcome if ethnic cannabis exposure terms were included as covariates in the model and no instrumental variables were used at all. This interesting and highly significant model is shown in the final panel of Table 2. Δ9THC exposure and the Δ9THC: cannabigerol interaction are both significant as are five ethnic cannabis exposure terms.

### Effect size

**Fig. 6:**
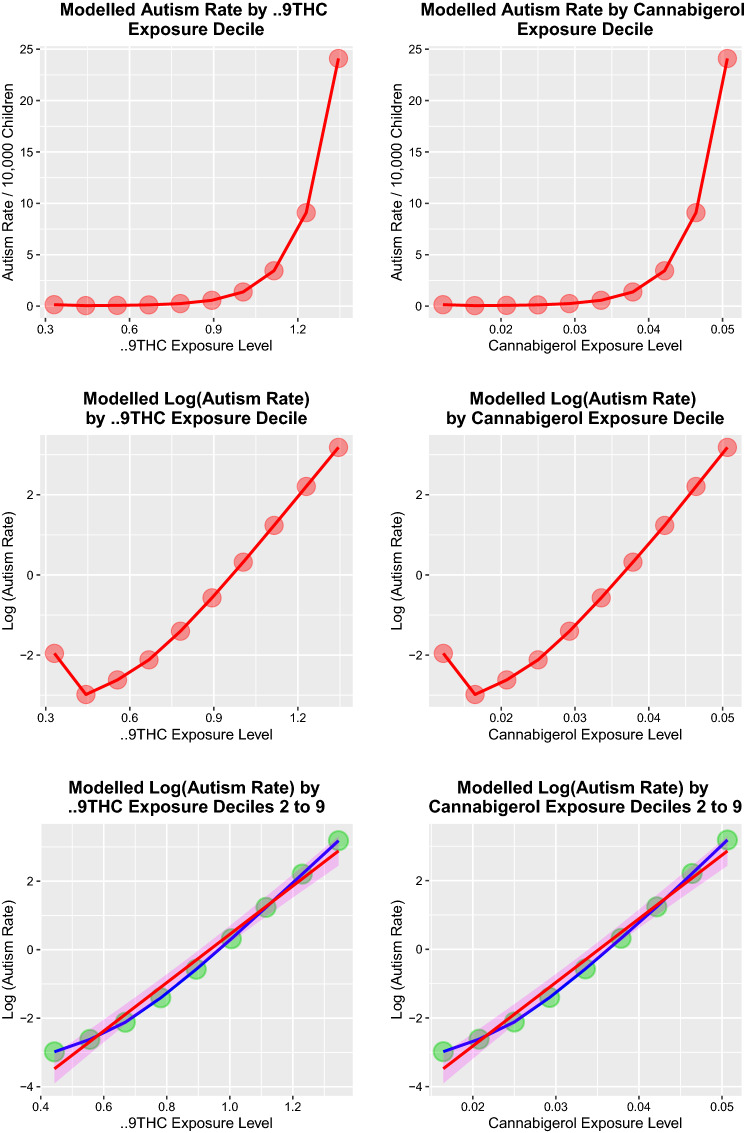
Modelled autism rate by exposure to Δ9THC and cannabigerol. **A** Linear. **B** Logarithmic and **C** regression plots for Δ9THC and cannabigerol, respectively

The availability of a final (unlagged) geospatial model allows modelling of cannabinoid effects and potentially the calculation of an effect size. When minimal and maximal values for THC and cannabigerol exposure are inserted into this model, autism rates of 0.37 and 38.42, respectively, are predicted, a variation of 102.72-fold. Similarly, ASMR at each decile of cannabinoid exposure may be calculated as shown in eTable 8 and Fig. 6. Steep rises with rising cannabinoid concentration are shown (top panels) which are linear on log plots, thus implying exponential relationships (middle panels) and to which tight-fitting regression lines may be fitted for deciles 2–9 (lower panels). The exponential regression coefficients for the relationship between ASMR and THC and cannabigerol exposure for deciles 2–9 are 7.053 (6.39–7.71) and 185.334 (167.88–202.79) with both *P* < 2.0 × 10^–7^ (eTable 9) and both Pearson correlation coefficients *R* > 0.992, *P* < 2.0 × 10^–7^.

As one doubles the THC exposure from 0.4 to 0.8 and to 1.6%% (compound units), the predicted ASMR rises from 0.022 to 0.382 to 107.83/10,000 children or 4,736.81-fold. As the cannabigerol exposure rises from 0.02 to 0.04 to 0.08%%, the modelled ASMR rises from 0.059 to 2.43 to 4029.65/10,000 children, or 67,511.42-fold which reflects the exponential relationship.

The THC–cannabigerol–autism rate relationship is illustrated from different perspectives in the 3D plots of eFigure 9.

### Causal inference

In addition to geospatiotemporal modelling, this dataset lends itself also to the techniques of formal causal inference to investigate further the nature of the association between cannabis exposure and autism.

Inverse probability weighting was conducted considering the monthly use of cannabis as the key exposure of interest. Although this was an observational ecological study, weighting the key exposure variable in this manner allows one to achieve a quasi-randomized design. Robust regression was conducted in the R package survey.

When a full list of the five drug variables, four ethnicities, median household income and five ethnic cannabis exposures was included in the robust regression model, the results are as shown in Table 3. In the additive model only a single ethnicity, non-Hispanic Asian is significant. The other five significant terms all include cannabis. Cannabis exposure alone is significant (*β* estimate = 1.08 (0.63–1.54), *P* = 2.90 × 10^–5^) and terms involving ethnic cannabis exposure are significant (from *β* estimate = 3.63 (2.94–4.34), *P* = 5.9 × 10^–13^).

**Table 3:** Multivariable robust regression models of autism rate

Parameter	Estimate	CI	*P* Value
Additive			
Hispanic.Cannabis	3.63	(2.94–4.34)	5.9E-13
NHAIAN.Cannabis	1.94	(1.34–2.55)	1.3E-07
NHAsian.Cannabis	1.27	(0.81–1.73)	2.3E-06
Cannabis	1.08	(0.63–1.54)	2.9E-05
NHAsian	0.25	(0.13–0.37)	2.0E-04
NHWhite.Cannabis	– 11.70	(– 16.61 to – 6.81)	2.8E-05
Interactive			
NHAsian	0.31	(0.15–0.47)	0.0008
Cannabis	803.00	(326.72–1279.28)	0.0024
Cannabis: Analgesics	265.20	(105.46–424.54)	0.0026
Analgesics	791.40	(302.96–1279.04)	0.0032
Cigarettes: Alcohol: Cannabis	32,850.00	(8008–57,792)	0.0145
Cigarettes: Alcohol: Cannabis: Analgesics	10,730.00	(2428.8–18,971.2)	0.0160
Cigarettes: Alcohol	97,700.00	(22,240–173,160)	0.0163
Cigarettes: Alcohol: Analgesics	31,900.00	(6812–56,988)	0.0184
NHWhite.Cannabis	– 3.98	(– 7.12 to – 0.84)	0.0185
Alcohol: Analgesics	– 9688.00	(– 16393.2 to – 2986.8)	0.0080
Cigarettes: Analgesics	– 2634.00	(– 4454.76 to – 805.24)	0.0080
Alcohol	– 29570.00	(– 49,788 to – 9412)	0.0071
Cigarettes	– 7994.00	(– 13419.2 to – 2560.8)	0.0070
Alcohol: Cannabis: Analgesics	– 3244.00	(– 5435.2 to – 1044.8)	0.0069
Cigarettes: Cannabis: Analgesics	– 886.60	(– 1484.8 to – 289.2)	0.0066
Alcohol: Cannabis	– 9894.00	(– 16534.4 to – 3245.6)	0.0063
Cigarettes: Cannabis	– 2689.00	(– 4477.52 to – 902.48)	0.0059

*CI* 95% confidence interval, *NH* non-Hispanic, *NHAIAN* non-Hispanic-American Indian/Alaskan-Native

In a model including a four-way interaction term between substance exposure terms tobacco–alcohol–cannabis–analgesics, 13 of 22 terms remaining in the final model included cannabis. In five cases, this related to ethnic cannabis exposure. In eight cases cannabis exposure itself was significant in interactive terms. Cannabis exposure alone was also significant (*β* estimate = 803.00 (326.72–1279.28), *P* = 0.0024).

When a similar exercise is conducted using mixed effects models, qualitatively similar results were obtained (eTable 10).

The above findings using IPW show that cannabis appears to be causally related to the autism rate. However, it is theoretically possible that some unidentified and unmeasured confounding factor, which is correlated with both the exposure of interest and the outcome, might be confounding these results in the background. The magnitude required of this unknown dual correlation effect to obviate the present results can be quantified using the eValue.

Table 4 lists a set of eValues calculated from some of the main results of this study listed above. One notes that many of these eValues are very high, especially those deriving from spatial models. This implies that a significant degree of unmeasured confounding is unlikely. This fits with the highly significant findings obtained in many of the earlier results, and particularly with the close geotemporospatial relationships demonstrated earlier.

**Table 4:** eValues for major results from foregoing analyses

Parameter	Table	*β* estimate (C.I.)	RR (95% CI)	eValues
Decile Estimates				
THC_Decile	eTable 9	7.0526 (6.37, 7.71)	1.10E + 27 (3.26E + 24, 3.71E + 29)	2.19E + 27, 6.51E + 24
CBG_Decile		185.33 (167.87, 202.79)	Infinity (Infinity, Infinity)	Infinity, Infinity
Mixed Effects Models				
Additive				
African.Am.Cannabis	eTable 10	0.509099 (0.39–0.63)	1.011 (1.008, 1.014)	1.12, 1.10
Cannabis		0.393926 (0.30–0.49)	1.102 (1.064, 1.011)	1.10, 1.08
NHAIAN.Cannabis		0.258642 (0.10–0.41)	1.006 (1.002, 1.009)	1.08, 1.05
Interactive				
Cigarettes: Cannabis: Cocaine	eTable 10	3753.1 (1451.28–6054.92)	6.1E + 51 (1.22E + 20, 3.04E + 83)	1.22E + 52, 4.5E + 20
Cigarettes: Cannabis		15,065.8 (5585.85–24,545.75)	7.6E + 207 (2.2E + 77, Infinity)	Infinity, 4.37E + 77
NHWhite.Cannabis		2 (0.73–3.27)	1.06 (1.02, 1.11)	1.33, 1.18
Cigarettes: Cannabis: Analgesics: Cocaine		1167.2 (409.64–1924.76)	1.3E + 16 (4.7E + 04, 3.4E + 26)	2.5E + 16, 9.4E + 05
Cigarettes: Cannabis: Analgesics		4717 (1593.82–7840.18)	1.2E + 65 (2.0E + 22, 1.2E + 108)	2.4E + 65, 2.4E + 22
Alcohol: Cannabis: Cocaine		9890.4 (2955.51–16,825.29)	2.9E + 136 (9.4E + 40, 9.3E + 2321)	5.9E + 136, 1.88E + 41
Alcohol: Cannabis		38,348.6 (9877.05–66,820.15)	Infinity (1.2E + 137, Infinity)	Infinity, 2.4E + 137
Alcohol: Cannabis: Analgesics: Cocaine		2955.3 (673.59–5237.01)	6.0E + 40 (2.3E + 09, 1.5E + 72)	1.2E + 41, 4.5E + 09
Alcohol: Cannabis: Analgesics		11,491.4 (2112.41–20,870.39)	3.6E + 158 (2.6E + 29, 5.2E + 287)	Infinity, 5.1E + 129
Spatial Spreml Models				
0 lags				
THC	Table 2	4.58 (1.41, 7.76)	1.92E + 15 (5.34E + 04, 6.93E + 25)	3.85E + 15, 1.07E + 04
Cannabigerol		0.81 (0.33, 1.29)	495.54 (12.81, 1.92E + 04)	990.59, 25.11
THC*Cannabigerol		0.94 (0.21, 1.67)	1.38E + 03(5.30, 3.61E + 04)	2.77E + 03, 10.07
Cigarettes: Cannabigerol: Alcohol		8.91 (2.80, 15.02)	4.82E + 29 (2.38E + 07, 9.75E + 49)	9.65E + 29, 4.77E + 09
2 lags				
THC	Table 2	1.14 (0.35, 4.31)	6.03E + 03 (14.51, 2.51E + 06)	1.21E + 04, 28.51
Cannabigerol		0.81 (0.23, 1.39)	480.0 (5.65, 4.07E + 04)	959.59, 10.78
THC*Cannabigerol		0.25 (0.023, 0.46)	6.48 (1.29, 32.42)	12.44 1.91
4 lags				
THC	Table 2	1.95 (0.87, 3.04)	349.01 (13.73, 8.87E + 04)	697.51, 26.95
THC*Cannabigerol		71.65 (25.41, 117.88)	1.19E + 93 (1.36E + 33, 1.05E + 153)	2.39E + 93, 2.71E + 33
0 Lags, Zero Instrumental Variables				
THC	Table 2	2.07 (1.23, 2.91)	5.71E + 06 (10.5E + 04, 3.11E + 09)	1.14E + 08, 2.10E + 04
Afrc.Am.Cannabis		0.29 (0.14, 0.44)	9.24 (3.04, 28.02)	17.97, 5.54
Hispanic.Am.Cannabis		2.95 (1.99, 3.93)	4.28E + 09 (3.04E + 06, 6.04E + 12)	8.56E + 10, 6.07E + 06
Asian.Am.Cannabis		2.6 (1.78, 3.42)	2.98E + 08 (6.60E + 05, 1.34E + 11)	5.96E + 08, 1.32E + 06
AIAN.Am.Cannabis		0.48 (0.32, 0.65)	37.14 (10.95, 125.96)	73.77, 21.39
THC: Cannabigerol		0.24 (0.06, 0.41)	5.82 (1.53, 22.12)	11.12, 2.43

*THC* Δ9 tetrahydrocannabinol, *CBG* cannabigerol, *Am* American, *NH* non-Hispanic, *RR* relative rate, *CI* 95% confidence interval

These 29 E-value estimates and lower bounds may be listed consecutively as shown in Table 5. Since both *E* Value lists are shown in descending order, this presentation disrupts the pairing structure shown in Table 4. From this table it is observed that of the E-value estimates, 4 are infinite and 25/30 (83.3%) exceed 9 and so are in the high range [40] and 26/30 (86.7%) are greater than 1.25 and thus exceed the threshold of causality [29]. Similarly for the minimum E-values, 1 is infinite, 22/30 (73.3%) exceed 9 and thus are in the high range and 25/30 exceed 1.25 (83.3%) and therefore cross the threshold for causal effects. Considering the descriptive statistics for these two data pairs, the E-value estimates have a median of 5.97 × 10^8^ (interquartile range (IQR) 17.97, 2.40 × 10^65^) and the lower bound of the E-values has a median value of 1.07 × 10^4^ (IQR 5.54, 6.51 × 10^24^). These are very high and very dramatic results and effectively exclude a significant role for hypothetical confounder covariates.

**Table 5:** List of E-Values

No	*E* Value Estimates	Lower Bound *E* Values
1	Infinity	Infinity
2	Infinity	2.40E + 137
3	Infinity	5.10E + 129
4	Infinity	4.37E + 77
5	5.90E + 136	1.88E + 41
6	2.39E + 93	2.71E + 33
7	2.40E + 65	6.51E + 24
8	1.22E + 52	2.40E + 22
9	1.20E + 41	4.50E + 20
10	9.65E + 29	4.77E + 09
11	2.19E + 27	4.50E + 09
12	2.50E + 16	6.07E + 06
13	3.85E + 15	1.32E + 06
14	8.56E + 10	9.40E + 05
15	5.96E + 08	2.10E + 04
16	1.14E + 08	1.07E + 04
17	1.21E + 04	28.51
18	2.77E + 03	26.95
19	990.59	25.11
20	959.59	21.39
21	697.51	10.78
22	73.77	10.07
23	17.97	5.54
24	12.44	2.43
25	11.12	1.91
26	1.33	1.18
27	1.12	1.10
28	1.10	1.08
29	1.08	1.05

Finally, it has previously been shown that liberal legislative paradigms for cannabis are associated with elevated rates of autism [41]; however, this has not been confirmed in the geospatial context. eFigure 11 shows the (log) autism rate against time by legal status dichotomized as illegal status v. liberal status. Table 6 sets out the result of geospatial regression of the (log) autism rate against the dichotomized legal status and confirms a highly significant finding. This regression coefficient is associated with a relative risk of 2.05 (95% CI 1.20, 3.49) and eValues of 3.51 and 1.70, which are clearly relatively high. These E-values have been included in Table 5.

**Table 6:** Geospatial Regression of Dichotomized Legal Status

Parameters				Model			
Parameter	Estimate	95% CI	*P* Value	LogLik	Parameters	Value	*P* value
Spatial spreml Model							
Liberal Legal Status	0.0938	(0.02–0.16)	0.0085	191.68	phi	9.8E-06	NA
					psi	0.9508	< 2.2e-16
					rho	– 0.8141	< 2.2e-16
					lambda	0.0938	< 2.2e-16

*CI* 95% Confidence Interval, *LogLik* Log likelihood ratio at model optimization

## Discussion

The principal question addressed by the present study was to explore the mystery of the remarkable rise in US autism rate which has remained hitherto largely unexplained. This study is an epidemiological investigation which uses national panel and state-level geospatial regression to analyse ecological covariates of childhood autism across a diverse range of domains including socioeconomic, ethnicity and drug exposure. A particular focus of this study is on environmental exposure to cannabis and selected cannabinoids which have been noted to be neurotoxic with effects on foetal brain development including microcephaly, anencephaly and impaired child neurological development [8, 42–45]. Given historically very different and well-established rates of cannabis use by ethnic groups, two-stage panel and geospatial regression techniques have been utilized to carefully adjust for these effects.

Spatiotemporal regression studies implicate both ethnic and drug exposure variables as being significantly associated with autism incidence with three ethnicities, Caucasian-American, Asian-American and American-Indian and Alaskan-Native Americans, three drugs, tobacco, alcohol abuse or dependence, and two cannabinoids, Δ9THC and cannabigerol, remaining in final models with high levels of statistical significance when ethnic cannabis use is included as instrumental variables. When ethnic cannabis use is included as covariates, all five of them remain significant in final models.

Application of the techniques of causal inference to this dataset indicate that the cannabis–autism association satisfies the criteria for causality.

Geospatial analysis confirmed the previously demonstrated increased rate of autism in states where cannabis is legal.

Of importance, effect size studies demonstrated that the relationship between both Δ9THC and cannabigerol and autism is exponential and powerful enough to induce the seismic paradigm shift which has been observed epidemiologically.

One notes also that autism is rising whilst the use of the classical intoxicants tobacco and alcohol is falling. Since opioid and cocaine use only impact a small segment of the community, this naturally impugns cannabis use which alone is rising dramatically.

Whilst the rise in autism rates has been said to be due to changes in its rate of diagnosis, careful studies in the USA have shown that the rise is indeed real beyond simply an increase in diagnostic suspicion or nosology [9].

Modelling studies based on the final models across both space and time provide robust epidemiological evidence of a strong upward exponential association between both Δ9THC and cannabigerol and the autism rate. Combined with concordant trends in tobacco, alcohol and cannabis use (mentioned above) and multiple biological pathways (mentioned below), and satisfaction of causal criteria, these strong and consistent findings across both space and time strongly implicate rising cannabis exposure in the community and in pregnancy as a primary underlying driver of the wave of autism and epidemiologically support our opening hypotheses.

Whilst cannabis was only used more than 3 days per month by 7.35% of the population in 2017, high intensity cannabis use has grown dramatically across the USA in the past decade with overall daily or near daily use doubling nationwide [39] and having increased from 0.38 to 1.5% in the > 35 years cohort 2002–2017 (Fig. 3 [12]). As part of increased use, the rate of cannabis exposure during the first trimester of pregnancy is growing steeply as cannabis use in the wider population increases. Furthermore, women are having their children later and in so doing are moving into older cohorts with cannabis users having a longitudinal history of greater cumulative cannabis exposure. It is noted due to the long half-time of cannabis retention and excretion from body fat stores in regular cannabis smokers that first trimester exposure will occur almost inevitably even if the mother stops cannabis consumption upon receiving a diagnosis of pregnancy [46, 47].

In this sense, therefore, the present rapid increase in numbers presenting with child autism is occurring against a background of sociodemographic trends in the wider community where high intensity cannabis use is becoming more common.

### Mechanisms

That cannabis potency and use is increasing, is retained in tissue for significant periods, and has been shown to have a number of severely neurotoxic activities particularly on the developing brain is pertinent. Several reports from CDC have linked cannabis exposure with anencephalus [43, 44] with separate data linking it to spina bifida in Canada [42], microcephaly in Hawaii [45] and adverse child neurological outcomes in Pittsburgh, Toronto and the Netherlands [8]. A generalized inhibitory effect on cell growth has been reported [48–51], as have interference with synapse formation by inhibition of neuroligin and neurexin, key partners in synapse formation and determination [7, 52, 53]; an uncoupling of neuronal mitochondrial oxidative phosphorylation [54, 55] and of grey–white matter connections [56], and increase in astrogliosis [47], neuroinflammation [57] and thus brain aging [58], an inhibition of brain neurogenesis and thus plasticity [59, 60] and adverse effect on the slit:robo ratio which is one of the key determinants of the formation of the exuberant cortex characterizing human beings [61, 62] along with numerous other genetic and epigenetic disruptions [63–66].

### Epigenetic mechanisms

Recently, profoundly insightful and deeply meaningful results from an epigenome-wide association study (EWAS) of cannabis dependence and withdrawal have been published [67]. The authors examined the DNA methylation status of 20 cannabis-dependent patients both before and after an 11-week period of documented abstinence and compared these results with those from a comparable group of cannabis-naïve control patients who were sampled at similar time points.

The results were of profound importance as relates to perturbation of normal brain development. Significant hits were found for the brain, cerebrum, cerebral cortex, head development, brain size, brain formation, forebrain patterning, proliferation of neural cells, brain neurogenesis, neuronal morphology, central nervous system development (139 hits), neuronal outgrowth and brain cell movement. When major brain receptors were considered, there were 132 hits for the AMPA receptor (GRIA), 165 hits for the kainate receptor (GRIK), 26 hits for the NMDA receptor (GRIN), 11 hits for the delta glutamate receptor (GRID), 122 hits for the metabotropic glutamate receptor (GRM), 125 hits for the GABA-A receptor (GABRA), 22 hits for the GABA-B receptor (GABRB), 85 hits for the serotonin receptor (HTR), 17 hits for the dopamine receptor (DRD1), 52 hits for the dopamine transporter (DAT, SLC6A3), 7 hits for the oxytocin receptor (OXTR), 5 hits for the μ-opioid receptor (ORPM1) and 5 hits for the δ-opioid receptor (ORPD1).

14 and 8 hits were noted for Down syndrome cell adhesion molecule (DSCAM) and discs large homolog associated protein 2 (DLGAP2) which have both been previously linked with autism [68–70].

As noted above, the exuberant outgrowth of the human cortex has been causally attributed to the slit–robo system. There were 351 hits for slits and 40 hits for robo. Additionally, there were 8 hits for a slit–robo Rho activating GTPase activating protein 2 (SRGAP2).

It has also been shown that the exuberant frontal outgrowth of the human cortex can be attributed to a steep gradient of the key human morphogen retinoic acid [71, 72]. A high concentration of this key transcription factor at the frontal pole fell to low levels at the premotor cortex. Indeed, forced expression of this gradient in the mouse reproduced the high number of cells seen in the human neocortex in the murine model [71]. The high frontal concentration of retinoic acid was maintained by an isoform of alcohol dehydrogenase (ALDH1), the lower premotor cortical level was controlled by metabolism by enzymes of the cytochrome system (CYP26B1) and the retinoic acid signal was transduced by the key retinoid receptors RXRA and RARB. There were 13 hits in the Schrott dataset for the enzymes of the ALDH1 system (including cadherin 8 and protocadherin 17), 10 hits for the cytochromes of the CYP2 series, and 9 hits for the retinoid receptor group.

While these very impressive and stunning results do not formally prove the salience of epigenomic results in the aetiology of cannabis-associated congenital brain damage, they do strongly imply that such data is highly pertinent and likely to at least partly contribute to meaningful and detailed explanatory and causal mechanisms which manifest clinically as the autistic spectrum of disorders.

### Causal inference

Some comments on the use of the techniques of causal inference in this study are in order. As mentioned in “Methods”, all mixed effects and robust regressions were performed with inverse probability weighting. This is the technique of choice in causal modelling, which has the effect of making an observational group broadly comparable across its subgroups, an effect which greatly increases the power of the study from being purely observational in nature to a pseudo-randomized study which has been shown to produce analytical results similar to those found in formal randomized controlled trials [73]. Hence the use of such inverse probability controlled modelling, especially using several regression techniques (here mixed effects and robust), allows us to be confident that the results reported are indeed of a causal nature and not simply associational as may otherwise be mistakenly assumed.

Secondly, we used the technique of E-values widely throughout the linear, mixed effects and spatial models which were reported. E-values quantitate the degree of association required of some hypothetical confounder covariate with *both* the exposure of interest and the outcome of concern to explain away an apparently causal relationship. The scale of the extraordinarily high E-values reported in this study is unprecedented in the autism literature to our knowledge. As noted in “Results”, we found that the median E-value estimate was very high 5.96 × 10^8^ and of the lower bound of the E-values was 1.07 × 10^4^. Five E-value estimates were infinite and one minimum E-value was infinite. E-values of this extremely high magnitude clearly discount the realistic possibility that the reported results may be due to some extraneous and unidentified confounder covariate [29, 30, 74–76]. It may be that the very high magnitude of the E-values reported in the present study reflect the very large sample size.

Combining inverse probability weighting, E-values, various forms of regression techniques along with the study of the association in its native space–time context provides several strong lines of analytical epidemiological evidence that the relationship reported is real in nature, powerful in its effect, and amply satisfies the quantitative criteria for epidemiological causality.

### Strengths and limitations

The present study has a number of strengths and limitations. Its strengths include the use of several nationally representative databases, the application of geospatial and causal inference analytical techniques to these questions for the first time to our knowledge, the timeliness of the information presented, the cultural and community-wide implications at a time when cannabis use is expanding rapidly the use of multiple forms of regression including space–time studies and the use of the formal and quantitative techniques of causal inferential modelling. The limitations of the present study relate mainly to its ecological design which include the lack of individual participant-level data. In the present study, community cannabis use was used as a surrogate marker for parental cannabis use, as there is no direct database of which we are aware which links these covariates directly, and as the cannabis use of pregnant women has been shown to follow community cannabis use in several studies [35, 36, 38, 77–79]. The findings of this exploration of these wide-ranging studies are, however, provocative and indicate further research in this area.

### Generalizability

Given that the data we have employed come from the USA, which by many metrics is reflective of other Western countries, the study findings are likely to be generalizable to other nations. Whilst there are to our knowledge no other similar wide-ranging analyses of autism, adverse reports of neurological function following widespread cannabis use have issued from other countries such as Egypt, China, India and Morocco [39].

## Conclusions

Our results implicate both Δ9THC and cannabigerol in these studies, which suggest that merely lowering the Δ9THC content of widely available cannabinoid preparations would not constitute a sufficient public health response. These data including geotemporospatial analysis and pseudo-randomization of an observational population confirm our opening hypothesis that increased cannabis use and its related socioethnodemographic trends is one of the principal causes and primary drivers of escalating US autism rates. The issue of the exponential relationship between exposure to the cannabinoids Δ9-tetrahydrocannabinol and cannabigerol is of particular concern and necessarily implies a non-linear, and in a public health sense, apparently abrupt relationship between exposure and downstream consequences, which would be consistent with multiple mechanistic pathways. In view of the present aggressive growth phase of the emerging cannabis industry, further research on the factors identified in this ecological study, including higher definition spatiotemporal epidemiological studies, are indicated.

## Supplementary Information

Below is the link to the electronic supplementary material.

Supplementary file1 (DOCX 100 KB)

Supplementary file2 (PDF 91 KB)

Supplementary file3 (PDF 48 KB)

Supplementary file4 (PDF 9 KB)

Supplementary file5 (PDF 35 KB)

Supplementary file6 (PDF 14 KB)

Supplementary file7 (PDF 27 KB)

Supplementary file8 (PDF 15 KB)

Supplementary file9 (PDF 2169 KB)

Supplementary file10 (PDF 5461 KB)

Supplementary file11 (PDF 37 KB)

## Data Availability

All data generated or analysed during this study are included in this published article and its supplementary information files. Data has been made publicly available on the Mendeley Database Repository and can be accessed from this URL http://dx.doi.org/10.17632/p7myt3fbzs.1
