# Supplementary material for: Impact of converging sociocultural and substance-related trends on US autism rates: combined geospatiotemporal and causal inferential analysis
Source: Eur Arch Psychiatry Clin Neurosci. 2022 Jul 2;273(3):699–717. doi: 10.1007/s00406-022-01446-0 (PMC10085966; doi:10.1007/s00406-022-01446-0)
Supplement: Supplementary file 1 — Supplementary file1 (DOCX 100 KB) [file 406_2022_1446_MOESM1_ESM.docx]

**Table of Contents**

**Supplementary Tables - eTables**

| **Item** | **eTables** | **Page No.** |
| --- | --- | --- |
|  |  |  |
| Text | Statistical Methods | 2 |
|  |  |  |
| eTable 1 | National Data Input | 3 |
| eTable 2 | National Patterns of Monthly Cannabis Smoking | 7 |
| eTable 3 | Autism Rate by Ethnicity, Interactive Time-Dependent Model | 8 |
| eTable 4 | Robustness Analysis with High and Low Forward Estimates of Autism Rates | 9 |
| eTable 5 | Spatiotemporal spreml Lagged Models with Drugs, Ethnicity and Socioeconomic Covariates | 11 |
| eTable 6 | Spatiotemporal spreml Lagged Models with Drugs, Ethnic Cannabis Use and Socioeconomic Covariates | 13 |
| eTable 7 | Full Geospatial Results Including Model Parameters | 15 |
| eTable 8 | Deciles of THC and Cannabigerol Exposure | 17 |
| eTable 9 | Predicted Model Outputs | 18 |
| eTable 10 | Weighted Mixed Effects Regression Models | 19 |

Statistical Methods

Programs used.

Data was processed using “R Studio” version 1.2.5042 based on “R” version 4.0.0 ^1^. Graphs were drawn with R package ggplot2 ^2^ and bivariate graphs were drawn using colorplaner ^3,4^. R package sf (“Simple Features”) was used for map drawing ^5^. Data was manipulated with dplyr amongst others ^3^. Multiple graphs were assembled with ggarrange from ggpubr ^6^. 3D graphs were drawn using NCSS software ^7^. Correlation tests were calculated using cor.test from R-package stats. For straightforward linear regression least squares regression was employed from R-Base. Panel regression was performed using R package plm ^8,9^. Function spreml from the splm package was used for geospatial analyses ^9-11^.

**References**

1. R-3.6.1 for Windows (32/64 bit). CRAN: Central "R" Archive Network, 2020. (Accessed March 5th 2020, 2020, at <https://cran.r-project.org/bin/windows/base/>.)

2. Wickham H. ggplot2: Elegant Graphics for Data Analysis. New York: Springer-Verlag; 2016.

3. Wickham H., Averick M., Bryan J., et al. Welcome to the Tidyverse. Journal of Open Source Software 2019;4:1686-91.

4. Colorplaner: ggplot2 Extension to Visualize Two Variables Per Color Aesthetic Through Colorspace Projection. Github, 2020. (Accessed October 6th 2020, 2020, at <https://github.com/wmurphyrd/colorplaner>.)

5. Pebesma E. Simple Features for R: Standardized Suport for Spatial Vector Data. The R Journal 2018;10:439-46.

6. ggpubr: 'ggplot2' Based Publication Ready Plots. R package version 0.4.0, 2020. (Accessed 12th December 2020, 2020, at <https://CRAN.R-project.org/package=ggpubr>.)

7. NCSS Statistical Software. NCSS, LLC, 2020. (Accessed 26th April 2020, 2020, at <https://www.ncss.com/>.)

8. Package 'plm'. CRAN: Central "R" Archive Network, 2014. (Accessed 7th May 2020, 2020, at <https://cran.r-project.org/web/packages/plm/plm.pdf>.)

9. Croissant Y., Millo G. Panel Data Econometrics with R. Hoboken, New JErsey, USA; West Sussex, U.K.: Wiley and Sons; 2019.

10. Millo G., Piras G. splm: Spatial Panel Data Models in R. Journal of Stastistical Software 2012;47:1-38.

11. Millo G., Piras G. Package 'splm'. <https://cran.r-project.org/web/packages/splm/splm.pdf> ed. Trieste, Italy: CRAN (Central R-Archive Network); 2018:1-27.

**eTable 1.: National Level Data Inputs**

| Year | AutRtNat | Cigarette Use, Monthly | Alcophol Use Disorder | Cannabis Use Last Month | Cannabis Use Last Year | Cannabis Incidence | TrimCanExp | Potency | MeanMonthly Days of Cannabis Use | Mean Days Cannabis Use * Potency Product | Cannabis Use First Trimester Pregnancy | Heroin Use, Annual | Cocaine Use Annual |
| --- | --- | --- | --- | --- | --- | --- | --- | --- | --- | --- | --- | --- | --- |
| 1994 | 6.9847 |  |  |  |  |  |  |  |  |  |  |  |  |
| 1995 | 8.7784 |  |  |  |  |  |  |  |  |  |  |  |  |
| 1996 | 10.5553 |  |  |  |  |  |  |  |  |  |  |  |  |
| 1997 | 12.9676 |  |  |  |  |  |  |  |  |  |  |  |  |
| 1998 | 17.0946 |  |  |  |  |  |  |  |  |  |  |  |  |
| 1999 | 21.6236 |  |  |  |  |  |  |  |  |  |  |  |  |
| 2000 | 27.4305 | 0.2662 |  |  |  |  |  | 4.8105 |  |  |  |  |  |
| 2001 | 31.0914 | 0.2631 |  |  |  |  |  | 5.2638 |  |  |  |  |  |
| 2002 | 36.0045 | 0.2726 | 0.0770 | 0.0620 | 0.1024 | 0.0220 | 0.0001 | 6.2960 | 0.1532 | 0.9647 | 0.0009 | 0.0020 | 0.0090 |
| 2003 | 41.0293 | 0.2670 | 0.0750 | 0.0620 | 0.1011 | 0.0200 | 0.0001 | 6.2296 | 0.1530 | 0.9533 | 0.0009 | 0.0010 | 0.0100 |
| 2004 | 45.7927 | 0.2648 | 0.0780 | 0.0610 | 0.1011 | 0.0210 | 0.0000 | 7.0486 | 0.1503 | 1.0593 | 0.0003 | 0.0020 | 0.0080 |
| 2005 | 52.8350 | 0.2662 | 0.0770 | 0.0600 | 0.1005 | 0.0210 | 0.0001 | 7.1042 | 0.1483 | 1.0535 | 0.0005 | 0.0020 | 0.0100 |
| 2006 | 61.0944 | 0.2631 | 0.0770 | 0.0600 | 0.0994 | 0.0210 | 0.0000 | 7.4347 | 0.1513 | 1.1249 | 0.0001 | 0.0020 | 0.0100 |
| 2007 | 67.8476 | 0.2575 | 0.0750 | 0.0580 | 0.0997 | 0.0210 | 0.0002 | 8.0399 | 0.1448 | 1.1640 | 0.0014 | 0.0020 | 0.0080 |
| 2008 | 76.9083 | 0.2523 | 0.0740 | 0.0610 | 0.1061 | 0.0220 | 0.0001 | 8.5237 | 0.1572 | 1.3403 | 0.0006 | 0.0020 | 0.0070 |
| 2009 | 85.8341 | 0.2471 | 0.0750 | 0.0670 | 0.1122 | 0.0240 | 0.0001 | 8.1207 | 0.1738 | 1.4114 | 0.0009 | 0.0020 | 0.0070 |
| 2010 | 92.9588 | 0.2407 | 0.0710 | 0.0690 | 0.1127 | 0.0240 | 0.0002 | 9.3977 | 0.1890 | 1.7766 | 0.0017 | 0.0020 | 0.0060 |
| 2011 | 103.3607 | 0.2397 | 0.0650 | 0.0700 | 0.1119 | 0.0260 | 0.0001 | 11.0107 | 0.1856 | 2.0438 | 0.0014 | 0.0020 | 0.0050 |
| 2012 | 108.8500 | 0.2368 | 0.0680 | 0.0730 | 0.1162 | 0.0240 | 0.0005 | 12.1051 | 0.2020 | 2.4452 | 0.0059 | 0.0030 | 0.0060 |
| 2013 | 119.9700 | 0.2333 | 0.0660 | 0.0750 | 0.1222 | 0.0240 | 0.0004 | 13.0000 | 0.2089 | 2.7162 | 0.0047 | 0.0030 | 0.0060 |
| 2014 | 131.6700 | 0.2269 | 0.0640 | 0.0840 | 0.1287 | 0.0260 | 0.0001 | 14.0000 | 0.2373 | 3.3226 | 0.0014 | 0.0030 | 0.0060 |
| 2015 | 143.9400 | 0.2172 | 0.0590 | 0.0830 | 0.1341 | 0.0260 | 0.0003 | 15.0000 | 0.2302 | 3.4528 | 0.0041 | 0.0030 | 0.0070 |
| 2016 | 156.7900 | 0.1910 | 0.0560 | 0.0890 | 0.1394 | 0.0260 | 0.0003 | 16.0000 | 0.2417 | 3.8666 | 0.0044 | 0.0040 | 0.0070 |
| 2017 | 170.2100 | 0.1790 | 0.0530 | 0.0960 | 0.1500 | 0.0300 | 0.0003 | 17.0000 | 0.2645 | 4.4964 | 0.0052 | 0.0030 | 0.0080 |
| 2018 | 184.2000 | 0.1730 | 0.0290 | 0.1010 | 0.1596 |  |  | 18.0000 |  |  |  | 0.0030 | 0.0080 |

| Year | US_MHY | d9_THC | CBD | CBC | CBN | CBG | THCV | White | Black | Hispanic | AIAN | Asian |
| --- | --- | --- | --- | --- | --- | --- | --- | --- | --- | --- | --- | --- |
| 1994 | 32264 | 3.5000 | 0.4600 | 0.2200 | 0.3200 |  |  | 0.7817 | 0.1215 | 0.1041 | 0.0082 | 0.0313 |
| 1995 | 34076 | 3.9600 | 0.2800 | 0.1900 | 0.3900 | 0.1360 | 0.0500 | 0.7764 | 0.1218 | 0.1077 | 0.0083 | 0.0321 |
| 1996 | 35492 | 4.5100 | 0.3700 | 0.2000 | 0.3800 | 0.1600 | 0.0900 | 0.7712 | 0.1220 | 0.1112 | 0.0083 | 0.0328 |
| 1997 | 37005 | 5.0100 | 0.4100 | 0.1900 | 0.3400 | 0.2000 | 0.1100 | 0.7659 | 0.1223 | 0.1148 | 0.0083 | 0.0336 |
| 1998 | 38885 | 4.9000 | 0.4100 | 0.2000 | 0.3800 | 0.1700 | 0.0500 | 0.7606 | 0.1225 | 0.1183 | 0.0084 | 0.0344 |
| 1999 | 40696 | 4.6000 | 0.4200 | 0.1700 | 0.5500 | 0.1700 | 0.0700 | 0.7553 | 0.1228 | 0.1219 | 0.0084 | 0.0352 |
| 2000 | 41990 | 5.3400 | 0.5200 | 0.1800 | 0.5100 | 0.2600 | 0.0800 | 0.7500 | 0.1230 | 0.1255 | 0.0085 | 0.0360 |
| 2001 | 42228 | 6.1100 | 0.5500 | 0.1900 | 0.4000 | 0.2900 | 0.0900 | 0.7380 | 0.1227 | 0.1289 | 0.0093 | 0.0374 |
| 2002 | 42409 | 7.2000 | 0.4700 | 0.2100 | 0.2800 | 0.2800 | 0.1000 | 0.7260 | 0.1224 | 0.1324 | 0.0101 | 0.0388 |
| 2003 | 43318 | 7.1500 | 0.4700 | 0.2200 | 0.2900 | 0.3300 | 0.0900 | 0.7140 | 0.1221 | 0.1358 | 0.0110 | 0.0402 |
| 2004 | 44334 | 8.1400 | 0.5100 | 0.2300 | 0.3500 | 0.4000 | 0.1000 | 0.7020 | 0.1218 | 0.1393 | 0.0118 | 0.0416 |
| 2005 | 46326 | 8.0100 | 0.4800 | 0.2600 | 0.3900 | 0.4000 | 0.0900 | 0.6900 | 0.1215 | 0.1427 | 0.0126 | 0.0430 |
| 2006 | 48201 | 8.7600 | 0.4300 | 0.2400 | 0.3300 | 0.4000 | 0.0900 | 0.6780 | 0.1212 | 0.1462 | 0.0135 | 0.0444 |
| 2007 | 50233 | 9.5800 | 0.4600 | 0.2400 | 0.3100 | 0.4400 | 0.1000 | 0.6660 | 0.1209 | 0.1496 | 0.0143 | 0.0458 |
| 2008 | 50303 | 9.9300 | 0.4100 | 0.2500 | 0.4100 | 0.3700 | 0.1000 | 0.6540 | 0.1206 | 0.1635 | 0.0151 | 0.0442 |
| 2009 | 49777 | 9.7500 | 0.3900 | 0.2400 | 0.4800 | 0.3300 | 0.1000 | 0.6420 | 0.1203 | 0.1682 | 0.0160 | 0.0453 |
| 2010 | 49276 | 10.3600 | 0.2800 | 0.2500 | 0.5000 | 0.3400 | 0.0800 | 0.6300 | 0.1200 | 0.1730 | 0.0168 | 0.0463 |
| 2011 | 50054 | 11.1300 | 0.2200 | 0.2500 | 0.4500 | 0.4200 | 0.0900 | 0.6278 | 0.1206 | 0.1742 | 0.0159 | 0.0470 |
| 2012 | 51017 | 12.3000 | 0.2000 | 0.2400 | 0.5500 | 0.4300 | 0.0900 | 0.6256 | 0.1212 | 0.1754 | 0.0151 | 0.0476 |
| 2013 | 51939 | 12.0200 | 0.1700 | 0.2700 | 0.5800 | 0.4700 | 0.1000 | 0.6234 | 0.1218 | 0.1766 | 0.0142 | 0.0483 |
| 2014 | 53657 | 11.8400 | 0.1500 | 0.2300 | 0.4500 | 0.4600 | 0.0900 | 0.6212 | 0.1224 | 0.1778 | 0.0133 | 0.0490 |
| 2015 | 56516 |  |  |  |  |  |  | 0.6190 | 0.1230 | 0.1790 | 0.0125 | 0.0497 |
| 2016 | 59039 |  |  |  |  |  |  | 0.6130 | 0.1236 | 0.1820 | 0.0081 | 0.0503 |
| 2017 | 61372 |  |  |  |  |  |  | 0.6080 | 0.1260 | 0.1850 | 0.0082 | 0.0530 |
| 2018 | 63179 |  |  |  |  |  |  | 0.6040 | 0.1270 | 0.1870 | 0.0083 | 0.0539 |

| Year | HIPI | NHWhite Cannabis Use | NHBlack Cannabis Use | Hispanic Cannabis Use | Asian Cannabis Use | AIAN Cannabis Use | NHPI Cannabis Use | Mixed Cannabis Use | AutRtHi | AutRtLo |
| --- | --- | --- | --- | --- | --- | --- | --- | --- | --- | --- |
| 1994 | 0.0010 |  |  |  |  |  |  |  | 8.25 | 5.68 |
| 1995 | 0.0011 |  |  |  |  |  |  |  | 10.8 | 7.81 |
| 1996 | 0.0011 |  |  |  |  |  |  |  | 13.92 | 10.25 |
| 1997 | 0.0012 |  |  |  |  |  |  |  | 17.6 | 13.01 |
| 1998 | 0.0012 |  |  |  |  |  |  |  | 21.85 | 16.09 |
| 1999 | 0.0013 |  |  |  |  |  |  |  | 26.66 | 19.49 |
| 2000 | 0.0013 |  |  |  |  |  |  |  | 32.03 | 23.21 |
| 2001 | 0.0014 |  |  |  |  |  |  |  | 37.97 | 27.24 |
| 2002 | 0.0014 |  |  |  |  |  |  |  | 44.47 | 31.59 |
| 2003 | 0.0015 | 1.5794 | 0.8455 | 0.5256 | 0.1614 | 1.6063 | 1.0104 | 1.3612 | 51.54 | 36.26 |
| 2004 | 0.0015 | 1.5303 | 0.8709 | 0.6278 | 0.1280 | 1.0936 | 0.9057 | 1.3352 | 59.17 | 41.24 |
| 2005 | 0.0015 | 1.5084 | 1.0182 | 0.4938 | 0.1914 | 1.4672 | 0.5331 | 0.9560 | 67.36 | 46.55 |
| 2006 | 0.0016 | 1.5508 | 1.0009 | 0.4605 | 0.3076 | 1.2733 | 0.7943 | 0.8965 | 76.12 | 52.17 |
| 2007 | 0.0016 | 1.4747 | 0.9299 | 0.5128 | 0.2392 | 0.9452 | 0.3812 | 1.3560 | 85.44 | 58.11 |
| 2008 | 0.0017 | 1.5827 | 1.1363 | 0.5254 | 0.2407 | 1.0386 | 0.6164 | 1.6943 | 95.33 | 64.36 |
| 2009 | 0.0017 | 1.7845 | 0.9773 | 0.6877 | 0.1777 | 1.6886 | 0.4164 | 1.6878 | 105.78 | 70.94 |
| 2010 | 0.0017 | 1.9158 | 1.2013 | 0.7967 | 0.2675 | 1.2857 | 0.3539 | 1.3279 | 116.79 | 77.83 |
| 2011 | 0.0018 | 1.8810 | 1.1152 | 0.8187 | 0.2792 | 1.0214 | 0.7672 | 1.6050 | 128.37 | 85.04 |
| 2012 | 0.0018 | 2.0573 | 1.2055 | 0.8428 | 0.2791 | 1.2480 | 0.9441 | 1.8583 | 140.51 | 92.56 |
| 2013 | 0.0018 | 2.1278 | 1.2093 | 0.8015 | 0.2032 | 1.5975 | 2.6829 | 2.7750 | 153.22 | 100.41 |
| 2014 | 0.0019 | 2.4165 | 1.5849 | 0.8717 | 0.3297 | 1.7084 | 2.2875 | 1.9568 | 166.49 | 108.57 |
| 2015 | 0.0019 | 2.3267 | 1.6011 | 0.9045 | 0.2799 | 1.9568 | 0.9221 | 1.9510 | 180.33 | 117.05 |
| 2016 | 0.0017 | 2.4382 | 1.5459 | 0.9908 | 0.3938 | 1.9022 | 1.0834 | 2.4339 | 194.72 | 125.85 |
| 2017 | 0.0018 | 2.6959 | 1.6664 | 1.0727 | 0.3467 | 2.2770 | 1.1743 | 2.3604 | 209.69 | 134.96 |
| 2018 | 0.0018 |  |  |  |  |  |  |  | 225.21 | 144.4 |

**eTable 2.: Last Month Cannabis Smoking Patterns – National Level**

| Year | Mean Days Cannabis Use | % Smoking Trivially - ≤3 Days / Month | Mean Days Smoked Trivially | % Smoking Non-Trivially - ≥3 Days / Month | Mean Days Smoked Non-Trivially |
| --- | --- | --- | --- | --- | --- |
|  |  |  |  |  |  |
| 2002 | 0.153 | 95.30% | 0.0114 | 4.68% | 0.248 |
| 2003 | 0.153 | 95.40% | 0.0124 | 4.57% | 0.247 |
| 2004 | 0.150 | 95.50% | 0.0123 | 4.47% | 0.242 |
| 2005 | 0.148 | 95.50% | 0.0113 | 4.49% | 0.240 |
| 2006 | 0.151 | 95.50% | 0.0121 | 4.46% | 0.244 |
| 2007 | 0.145 | 95.80% | 0.0119 | 4.25% | 0.233 |
| 2008 | 0.157 | 95.50% | 0.0119 | 4.55% | 0.254 |
| 2009 | 0.174 | 94.90% | 0.0120 | 5.10% | 0.282 |
| 2010 | 0.189 | 94.60% | 0.0123 | 5.36% | 0.307 |
| 2011 | 0.186 | 94.70% | 0.0131 | 5.30% | 0.301 |
| 2012 | 0.202 | 94.30% | 0.0123 | 5.69% | 0.328 |
| 2013 | 0.209 | 94.20% | 0.0134 | 5.81% | 0.339 |
| 2014 | 0.237 | 93.40% | 0.0143 | 6.60% | 0.386 |
| 2015 | 0.230 | 93.60% | 0.0142 | 6.44% | 0.374 |
| 2016 | 0.242 | 93.20% | 0.0151 | 6.78% | 0.393 |
| 2017 | 0.264 | 92.60% | 0.0159 | 7.35% | 0.430 |

**eTable 3.: Autism Rate by Ethnicity, Interactive Time-Dependent Model**

| **Parameter** | **Parameter** | | | **Model Parameters** | | | |
| --- | --- | --- | --- | --- | --- | --- | --- |
|  | **Estimate** | **95% C.I.** | **Pr(>\|t\|)** | **R-Squared** | **F** | **dF** | **P** |
|  |  |  |  |  |  |  |  |
| ***lm(log(Rate)~poly(Year,degree=2)*Race*** |  |  |  |  |  |  |  |
| American Indian / Alaksan Native | 1.22 | (1.00-1.44) | < 2e-16 | 0.812 | 23.44 | 20,84 | 6.14E-26 |
| Mixed_Race | 1.45 | (1.23-1.67) | < 2e-16 |  |  |  |  |
| NHWhite | 1.67 | (1.45-1.89) | < 2e-16 |  |  |  |  |
| NHBlack | 0.94 | (0.72-1.16) | 1.1E-13 |  |  |  |  |
| Native Hawaiian / Pacific Islander | 0.74 | (0.52-0.96) | 6.9E-10 |  |  |  |  |
| Hispanic | 0.47 | (0.25-0.69) | 2.3E-05 |  |  |  |  |
| poly(Year, degree = 2)1:Mixed_Race | 3.77 | (1.65-5.89) | 8.0E-04 |  |  |  |  |
| poly(Year, degree = 2)1:NHWhite | 3.21 | (1.09-5.33) | 0.004 |  |  |  |  |
| poly(Year, degree = 2)1:Native Hawaiian / PI | 2.30 | (0.18-4.42) | 0.037 |  |  |  |  |
| poly(Year, degree = 2)2:American Indian / AN | 2.17 | (0.05-4.29) | 0.049 |  |  |  |  |
| poly(Year, degree = 2)1:NHBlack | 2.00 | (-0.12-4.12) | 0.068 |  |  |  |  |
| poly(Year, degree = 2)1:American Indian / AN | 1.89 | (-0.23-4.01) | 0.085 |  |  |  |  |

**eTable 4.: Sensitivity Analysis**

| **Instrumental Variables** | **Parameter** | **Parameter** | | | **Model** | | | |
| --- | --- | --- | --- | --- | --- | --- | --- | --- |
|  |  | **Estimate** | **95% C.I.** | **Pr(>\|t\|)** | **Adj. R-Squ** | **ChiSq** | **dF** | **P** |
|  |  |  |  |  |  |  |  |  |
|  |  |  |  |  |  |  |  |  |
| ***DAILY CANNABIS USE RATES*** |  |  |  |  |  |  |  |  |
| ***Low Estimates*** |  |  |  |  |  |  |  |  |
| ***2 Lags, 1 Interactions*** |  |  |  |  |  |  |  |  |
| lag(Cannabis, Monthly), 0:2 | Black Ethnicity | 12.0930 | (12.09-12.09) | < 2.2e-16 | 1.0000 | 2.6x10^11^ | 8 | < 2.2e-16 |
| lag(Δ9THC_Exposure), 0:2 | Median Household Income | 7.5E-06 | (7.5E-6-7.5E-6) | < 2.2e-16 |  |  |  |  |
| lag(Cannabigerol_Exposure), 0:2 | Cigarettes, Monthly | 4.4978 | (4.49-4.5) | < 2.2e-16 |  |  |  |  |
| Cocaine_Annual | Hispanic Ethnicity | 0.1171 | (0.12-0.12) | < 2.2e-16 |  |  |  |  |
|  | Mean_Days_Cannabis_Potency | 0.2055 | (0.2-0.21) | < 2.2e-16 |  |  |  |  |
|  | Analgesics, Annual | 0.1588 | (0.16-0.16) | < 2.2e-16 |  |  |  |  |
|  | Cigarettes: Mean_Days_Cannabis_Potency | -0.9112 | (-0.91--0.91) | < 2.2e-16 |  |  |  |  |
|  | White Ethnicity | -8.1359 | (-8.14--8.13) | < 2.2e-16 |  |  |  |  |
|  |  |  |  |  |  |  |  |  |
| ***High Estimates*** |  |  |  |  |  |  |  |  |
| ***2 Lags, 1 Interactions*** |  |  |  |  |  |  |  |  |
| lag(Cannabis, Monthly), 0:2 | Black Ethnicity | 12.8670 | (12.85-12.89) | < 2.2e-16 | 1.0000 | 1.3x10^9^ | 8 | < 2.2e-16 |
| lag(Δ9THC_Exposure), 0:2 | Median Household Income | 8.2E-06 | (8.2E-06-8.2E-06) | < 2.2e-16 |  |  |  |  |
| lag(Cannabigerol_Exposure), 0:2 | Cigarettes, Monthly | 4.8841 | (4.82-4.95) | < 2.2e-16 |  |  |  |  |
| Cocaine_Annual | Hispanic Ethnicity | 0.1297 | (0.13-0.13) | < 2.2e-16 |  |  |  |  |
|  | Mean_Days_Cannabis_Potency | 0.2185 | (0.21-0.23) | < 2.2e-16 |  |  |  |  |
|  | Analgesics, Annual | 0.1762 | (0.17-0.19) | < 2.2e-16 |  |  |  |  |
|  | Cigarettes: Mean_Days_Cannabis_Potency | -0.9664 | (-1.01--0.92) | < 2.2e-16 |  |  |  |  |
|  | White Ethnicity | -8.7028 | (-8.72--8.69) | < 2.2e-16 |  |  |  |  |
|  |  |  |  |  |  |  |  |  |
|  |  |  |  |  |  |  |  |  |
| ***FIRST TRIMESTER PREGNANCY DAILY CANNABIS USE RATES*** | | | | |  |  |  |  |
| ***Low Estimates*** |  |  |  |  |  |  |  |  |
| ***2 Lags, 1 Interactions*** |  |  |  |  |  |  |  |  |
| lag(Cannabis, Monthly), 0:2 | First_Trimester_Cannabis_Exposure | 0.1400 | (0.11-0.17) | < 2.2e-16 | 0.9984 | 5727.8 | 4 | < 2.2e-16 |
| lag(Δ9THC_Exposure), 0:2 | First_Trimester_Cannabis_Exposure: THC_Potency | -0.0651 | (-0.07--0.06) | < 2.2e-16 |  |  |  |  |
| lag(Cannabigerol_Exposure), 0:2 | White Ethnicity | -5.0199 | (-5.71--4.33) | < 2.2e-16 |  |  |  |  |
| Cocaine_Annual | Cocaine, Annual | 0.1935 | (0.12-0.26) | 7.12E-08 |  |  |  |  |
|  |  |  |  |  |  |  |  |  |
| ***High Estimates*** |  |  |  |  |  |  |  |  |
| ***2 Lags, 1 Interactions*** |  |  |  |  |  |  |  |  |
| lag(Cannabis, Monthly), 0:2 | First_Trimester_Cannabis_Exposure | 0.1484 | (0.12-0.18) | < 2.2e-16 | 0.9978 | 5702.4 | 4 | < 2.2e-16 |
| lag(Δ9THC_Exposure), 0:2 | First_Trimester_Cannabis_Exposure: THC_Potency | -0.0690 | (-0.08--0.06) | < 2.2e-16 |  |  |  |  |
| lag(Cannabigerol_Exposure), 0:2 | White Ethnicity | -5.3810 | (-6.12--4.64) | < 2.2e-16 |  |  |  |  |
| Cocaine_Annual | Cocaine, Annual | 0.2041 | (0.13-0.28) | 1.07E-07 |  |  |  |  |

**eTable 5.: Spatiotemporal spreml Lagged Models with Drugs, Ethnicity and Socioeconomic Covariates**

| **General** | **Parameters** | | | | **Model** | | | |
| --- | --- | --- | --- | --- | --- | --- | --- | --- |
| **Instumental** + **L Lagged Variables** | **Parameter** | **Estimate** | **95% C.I.** | **P-Value** | **LogLik** | **Parameters** | **Value** | **P-Value** |
|  |  |  |  |  |  |  |  |  |
| mrjmon | ***0 Lags*** |  |  |  |  |  |  |  |
| Δ9THC | NHWhite Ethnicity | 2.20 | (1.7-2.7) | < 2.2e-16 | 220.313 | phi | 5.7E-07 | 1 |
| Cannabigerol | NHAsian Ethnicity | 0.47 | (0.38-0.55) | < 2.2e-16 |  | psi | 0.9396 | < 2.2e-16 |
| NHWhite_Score | NHAIAN Ethnicity | -0.05 | (-0.07--0.02) | 7.6E-05 |  | rho | -0.5652 | 4.8E-08 |
| NHBlack_Score | Abuse/Dependence_Alcohol | -30.58 | (-51.74--9.42) | 0.005 |  | lambda | 0.5374 | < 2.2e-16 |
| Hispanic_Score | Cannabis_Monthly: Abuse/Dependence_Alcohol | -10.59 | (-18.15--3.02) | 0.006 |  |  |  |  |
| NHAsian_Score | Cannabis_Monthly | 0.76 | (0.15-1.37) | 0.015 |  |  |  |  |
| NHAIAN_Score |  |  |  |  |  |  |  |  |
|  |  |  |  |  |  |  |  |  |
| mrjmon,0:2 | ***2 Lags*** |  |  |  |  |  |  |  |
| Δ9THC, 0:2 | NHWhite Ethnicity | 2.20 | (1.62-2.77) | 6.0E-14 | 172.681 | phi | 5.1E-06 | NA |
| Cannabigerol, 0:2 | NHAsian Ethnicity | 0.40 | (0.29-0.51) | 3.3E-13 |  | psi | 0.9415 | NA |
| NHWhite_Score, 0:2 | NHAIAN Ethnicity | -0.05 | (-0.07--0.02) | 5.1E-05 |  | rho | -0.5333 | 5.2E-06 |
| NHBlack_Score, 0:2 | Cigarettes :Cannabis_Monthly :Abuse/Dependence_Alcohol | 8.41 | (3.08-13.74) | 0.002 |  | lambda | 0.5042 | 1.36E-14 |
| Hispanic_Score, 0:2 | NHBlack Ethnicity | 0.11 | (0.03-0.19) | 0.005 |  |  |  |  |
| NHAsian_Score, 0:2 | Cannabis_Monthly:Abuse/Dependence_Alcohol | -2.38 | (-4.07--0.68) | 0.006 |  |  |  |  |
| NHAIAN_Score, 0:2 |  |  |  |  |  |  |  |  |
|  |  |  |  |  |  |  |  |  |
| mrjmon,0:3 | ***3 Lags*** |  |  |  |  |  |  |  |
| Δ9THC, 0:3 | NHWhite Ethnicity | 2.36 | (1.74-2.98) | 9.0E-14 | 148.126 | phi | 1.7E-05 | 1.0000 |
| Cannabigerol, 0:3 | NHAsian Ethnicity | 0.46 | (0.33-0.58) | 4.2E-13 |  | psi | 0.9485 | < 2.2e-16 |
| NHWhite_Score, 0:3 | NHAIAN Ethnicity | -0.05 | (-0.07--0.03) | 9.4E-06 |  | rho | -0.4960 | 0.0002 |
| NHBlack_Score, 0:3 | Cigarettes | -12.23 | (-21.63--2.82) | 0.011 |  | lambda | 0.4458 | 1.6E-08 |
| Hispanic_Score, 0:3 | Cigarettes :Cannabis_Monthly | -3.87 | (-7.29--0.45) | 0.026 |  |  |  |  |
| NHAsian_Score, 0:3 | NHBlack Ethnicity | 0.09 | (0.01-0.17) | 0.027 |  |  |  |  |
| NHAIAN_Score, 0:3 | Cannabis_Monthly | 0.91 | (0.04-1.78) | 0.040 |  |  |  |  |

**eTable 6.: Spatiotemporal spreml Lagged Models with Drugs, Ethnic Cannabis Use and Socioeconomic Covariates**

| **General** | **Parameters** | | | | **Model** | | | |
| --- | --- | --- | --- | --- | --- | --- | --- | --- |
| **Instumental** + **L Lagged Variables** | **Parameter** | **Estimate** | **95% C.I.** | **P-Value** | **LogLik** | **Parameters** | **Value** | **P-Value** |
|  |  |  |  |  |  |  |  |  |
|  | ***0 Lags*** |  |  |  |  |  |  |  |
| Cannabis, Monthly,0:2 | Median Household Income | 0.454 | (0.28-0.63) | 5.1E-07 | 193.321 | phi | 1.5E-05 | 1 |
| Δ9THC, 0:2 | Ethnic_Cannabis_Exposure_Score | 0.177 | (0.09-0.26) | 4.6E-05 |  | psi | 0.9426 | < 2.2e-16 |
| Cannabigerol, 0:2 | Cigarettes: Abuse/Dependence_Alcohol | -55.483 | (-88.24--22.72) | 0.001 |  | rho | -0.7467 | < 2.2e-16 |
| NHWhite_Score | Cannabis, Monthly: Abuse/Dependence_Alcohol | -4.851 | (-8.01--1.69) | 0.003 |  | lambda | 0.6971 | < 2.2e-16 |
| NHBlack_Score | Cannabis, Monthly | 0.631 | (0.13-1.13) | 0.014 |  |  |  |  |
| Hispanic_Score | Cigarettes: Cannabis, Monthly | -1.064 | (-2.04--0.09) | 0.033 |  |  |  |  |
| NHAsian_Score |  |  |  |  |  |  |  |  |
| NHAIAN_Score |  |  |  |  |  |  |  |  |
|  |  |  |  |  |  |  |  |  |
|  | ***2 Lags*** |  |  |  |  |  |  |  |
| Cannabis, Monthly,0:2 | Cigarettes: Cannabis, Monthly: Abuse/Dependence_Alcohol | 10.881 | (5.97-15.79) | 1.4E-05 | 144.755 | phi | 2.9E-05 | 1 |
| Δ9THC, 0:2 | Cannabis, Monthly: Abuse/Dependence_Alcohol | -2.654 | (-4.27--1.04) | 0.001 |  | psi | 0.9465 | < 2.2e-16 |
| Cannabigerol, 0:2 | Ethnic_Cannabis_Exposure_Score | 0.136 | (0.05-0.22) | 0.002 |  | rho | -0.7320 | 2.0E-12 |
| NHWhite_Score, 0:2 | MHInc | 0.326 | (0.12-0.53) | 0.002 |  | lambda | 0.6938 | 2.2E-16 |
| NHBlack_Score, 0:2 |  |  |  |  |  |  |  |  |
| Hispanic_Score, 0:2 |  |  |  |  |  |  |  |  |
| NHAsian_Score, 0:2 |  |  |  |  |  |  |  |  |
| NHAIAN_Score, 0:2 |  |  |  |  |  |  |  |  |
|  |  |  |  |  |  |  |  |  |
|  | ***4 Lags*** |  |  |  |  |  |  |  |
| Cannabis, Monthly,0:4 | Ethnic_Cannabis_Exposure_Score | 0.161 | (0.07-0.26) | 0.001 | 97.4994 | phi | 9.5941 | NA |
| Δ9THC, 0:4 | Cannabis, Monthly: Abuse/Dependence_Alcohol | -2.587 | (-4.59--0.58) | 0.012 |  | psi | 0.7824 | < 2.2e-16 |
| Cannabigerol, 0:2 | Cannabis, Monthly: Abuse/Dependence_Alcohol: Cigarettes | 10.270 | (3.91-16.63) | 0.002 |  | rho | -0.7340 | 4.7E-08 |
| NHWhite_Score, 0:4 |  |  |  |  |  | lambda | 0.6594 | < 2.2e-16 |
| NHBlack_Score, 0:4 |  |  |  |  |  |  |  |  |
| Hispanic_Score, 0:4 |  |  |  |  |  |  |  |  |
| NHAsian_Score, 0:4 |  |  |  |  |  |  |  |  |
| NHAIAN_Score, 0:4 |  |  |  |  |  |  |  |  |

**eTable 7.: Final Geospatial Regression Models Including Model Parameters**

| **General** | **Parameters** | | | | **Model** | | | |
| --- | --- | --- | --- | --- | --- | --- | --- | --- |
| **Instumental** + **Lagged Variables** | **Parameter** | **Estimate** | **95% C.I.** | **P-Value** | **LogLik** | **Parameters** | **Value** | **P-Value** |
|  |  |  |  |  |  |  |  |  |
| ***0 Lags*** |  |  |  |  |  |  |  |  |
| Cannabis, Monthly | NHAsian Ethnicity | 0.43 | (0.33-0.53) | < 2.2e-16 | 233.366 | phi | 1.6E-07 | NA |
| Δ9THC | NHWhite Ethnicity | 2.01 | (1.42-2.6) | 1.5E-11 |  | psi | 0.9386 | < 2.2e-16 |
| Cannabigerol | Cannabigerol: Alcohol_Abuse | -13.77 | (-19.41--8.13) | 1.8E-06 |  | rho | -0.6020 | 2.2E-09 |
| NHWhite_Score | Alcohol_Abuse | -44.35 | (-65.89--22.81) | 5.5E-05 |  | lambda | 0.5000 | < 2.2e-16 |
| NHBlack_Score | Cannabigerol | 0.81 | (0.34-1.28) | 9.0E-04 |  |  |  |  |
| Hispanic_Score | NHAIAN Ethnicity | -0.04 | (-0.06--0.02) | 0.002 |  |  |  |  |
| NHAsian_Score | Cigarettes: Cannabigerol: Alcohol_Abuse | 8.91 | (2.79-15.03) | 0.004 |  |  |  |  |
| NHAIAN_Score | Δ9THC | 4.59 | (1.41-7.77) | 0.005 |  |  |  |  |
|  | Cigarettes: Δ9THC | -16.23 | (-28.64--3.82) | 0.010 |  |  |  |  |
|  | Δ9THC: Cannabigerol | 0.94 | (0.21-1.67) | 0.011 |  |  |  |  |
|  | Cigarettes: Δ9THC: Cannabigerol | -3.39 | (-6.21--0.57) | 0.018 |  |  |  |  |
|  |  |  |  |  |  |  |  |  |
|  |  |  |  |  |  |  |  |  |
| ***2 Lags*** |  |  |  |  |  |  |  |  |
| Cannabis, Monthly,0:2 | NHAsian Ethnicity | 0.42 | (0.3-0.54) | 3.1E-12 | 178.8988 | phi | 0.0000 | 1.0000 |
| Δ9THC, 0:2 | NHWhite Ethnicity | 1.95 | (1.22-2.68) | 1.2E-07 |  | psi | 0.9378 | < 2.2e-16 |
| Cannabigerol, 0:2 | Alcohol_Abuse | -43.92 | (-69.97--17.87) | 0.001 |  | rho | -0.4350 | 0.0011 |
| NHWhite_Score, 0:2 | NHAIAN Ethnicity | -0.06 | (-0.1--0.02) | 0.001 |  | lambda | 0.4158 | 5.4E-07 |
| NHBlack_Score, 0:2 | Cannabigerol: Alcohol_Abuse | -11.24 | (-18.12--4.36) | 0.001 |  |  |  |  |
| Hispanic_Score, 0:2 | Δ9THC | 1.14 | (0.36-1.92) | 0.005 |  |  |  |  |
| NHAsian_Score, 0:2 | Cannabigerol | 0.81 | (0.22-1.4) | 0.007 |  |  |  |  |
| NHAIAN_Score, 0:2 | Δ9THC: Cannabigerol | 0.25 | (0.03-0.47) | 0.023 |  |  |  |  |
|  | NHAfrican-American Ethnicity | 0.08 | (0-0.16) | 0.046 |  |  |  |  |
|  |  |  |  |  |  |  |  |  |
|  |  |  |  |  |  |  |  |  |
|  |  |  |  |  |  |  |  |  |
|  |  |  |  |  |  |  |  |  |
| ***4 Lags*** |  |  |  |  |  |  |  |  |
| Cannabis, Monthly,0:4 | NHAIAN Ethnicity | -0.11 | (-0.13--0.09) | 9.0E-15 | 132.859 | phi | 7.3530 | 0.0002 |
| Δ9THC, 0:4 | NHAsian Ethnicity | 0.37 | (0.23-0.51) | 1.9E-07 |  | psi | 0.8793 | < 2.2e-16 |
| Cannabigerol, 0:4 | NHWhite Ethnicity | 1.52 | (0.74-2.3) | 1.0E-04 |  | rho | -0.4960 | 0.0011 |
| NHWhite_Score, 0:4 | Cannabigerol: Alcohol_Abuse | -22.68 | (-34.89--10.47) | 3.0E-04 |  | lambda | 0.3196 | 0.0016 |
| NHBlack_Score, 0:4 | Δ9THC | 1.96 | (0.88-3.04) | 4.0E-04 |  |  |  |  |
| Hispanic_Score, 0:4 | Alcohol_Abuse | -72.45 | (-114.28--30.62) | 7.0E-04 |  |  |  |  |
| NHAsian_Score, 0:4 | Cigarettes: Cannabigerol: Alcohol_Abuse | 71.65 | (25.41-117.89) | 0.002 |  |  |  |  |
| NHAIAN_Score, 0:4 | Cigarettes: Δ9THC | -6.44 | (-10.63--2.25) | 0.003 |  |  |  |  |
|  | Cigarettes: Alcohol_Abuse | 214.56 | (56.98-372.14) | 0.008 |  |  |  |  |

Technical Notes:

logLik Log (Likelihood ratio) at model optimization

phi: idiosyncratic component of the spatial error term

psi: individual time-invariant component of the spatial error term

rho: spatial autoregressive parameter

lambda: spatial autocorrelation coefficient

**eTable 8.: Autism Rate by Deciles of Cannabinoid Exposure**

| **Decile** | **THC Exposure Decile** | **Cannabigerol Exposure Decile** | **Log (Autism Rate)** | **Autism Rate** |
| --- | --- | --- | --- | --- |
| **1** | 0.3310 | 0.0122 | -1.9570 | 0.1413 |
| **2** | 0.4435 | 0.0165 | -2.9822 | 0.0507 |
| **3** | 0.5560 | 0.0208 | -2.6186 | 0.0729 |
| **4** | 0.6685 | 0.0250 | -2.1174 | 0.1203 |
| **5** | 0.7810 | 0.0293 | -1.4031 | 0.2458 |
| **6** | 0.8935 | 0.0336 | -0.5715 | 0.5647 |
| **7** | 1.0050 | 0.0379 | 0.3198 | 1.3768 |
| **8** | 1.1150 | 0.0422 | 1.2359 | 3.4413 |
| **9** | 1.2300 | 0.0464 | 2.2076 | 9.0938 |
| **10** | 1.3450 | 0.0507 | 3.1820 | 24.0940 |

**eTable 9.: Linear Regression Models by Cannabinoid Exposure**

| **Parameter** | **Parameter** | | | **Model Parameters** | | | |
| --- | --- | --- | --- | --- | --- | --- | --- |
|  | **Estimate** | **CI** | **Pr(>\|t\|)** | **Adj. R-Squared** | **F** | **dF** | **P** |
|  |  |  |  |  |  |  |  |
| ***ΔTHC_Exposure*** |  |  |  |  |  |  |  |
| Intercept | -6.604 | (-7.23--5.98) | 1.5E-07 | 0.9820 | 437.7 | 1,7 | 1.4E-07 |
| THC_Decile | 7.053 | (6.39-7.71) | 1.4E-07 |  |  |  |  |
|  |  |  |  |  |  |  |  |
| ***Cannabigerol_Exposure*** |  |  |  |  |  |  |  |
| Intercept | -6.525 | (-7.14--5.91) | 1.5E-07 | 0.9818 | 432.9 | 1,7 | 1.5E-07 |
| Cannabigerol_Decile | 185.334 | (167.88-202.79) | 1.5E-07 |  |  |  |  |

**eTable 10.: Weighted Mixed Effects Regression Models**

| **Parameter** | **Value** | **C.I.** | **P-Value** | **AIC** | **BIC** | **logLik** |
| --- | --- | --- | --- | --- | --- | --- |
|  |  |  |  |  |  |  |
| ***Additive*** |  |  |  |  |  |  |
| NHAsian | 2.25 | (2.1-2.4) | 0.0000 | 1635.9 | 1701.142 | -801.9501 |
| Cigarettes | 10.03 | (8.42-11.64) | 0.0000 |  |  |  |
| NHWhite | 7.36 | (5.9-8.81) | 0.0000 |  |  |  |
| NHAfric.Am | 0.42 | (0.32-0.51) | 0.0000 |  |  |  |
| NHAfric.Am.Cannabis | 0.51 | (0.39-0.63) | 0.0000 |  |  |  |
| Cannabis | 0.39 | (0.3-0.49) | 0.0000 |  |  |  |
| NHAIAN.Cannabis | 0.26 | (0.1-0.41) | 0.0011 |  |  |  |
| Alcohol | 4.23 | (1.65-6.81) | 0.0014 |  |  |  |
| NHAsian.Cannabis | -0.35 | (-0.53--0.18) | 0.0001 |  |  |  |
| NHWhite.Cannabis | -1.18 | (-1.56--0.8) | 0.0000 |  |  |  |
| Median.Household.Income | -1.02 | (-1.23--0.81) | 0.0000 |  |  |  |
| Analgesics | -1.05 | (-1.25--0.85) | 0.0000 |  |  |  |
| NHAIAN | -0.29 | (-0.32--0.27) | 0.0000 |  |  |  |
|  |  |  |  |  |  |  |
| ***Interactive*** |  |  |  |  |  |  |
| NHAsian | 2.2 | (2.02-2.38) | 0.0000 | 1161.311 | 1333.9 | -537.6553 |
| NHWhite | 6 | (4.51-7.49) | 0.0000 |  |  |  |
| Cigarettes: Cocaine | 11011.7 | (4362.11-17661.29) | 0.0013 |  |  |  |
| Cigarettes: Cannabis: Cocaine | 3753.1 | (1451.28-6054.92) | 0.0015 |  |  |  |
| Cigarettes | 44159.3 | (16925-71393.6) | 0.0016 |  |  |  |
| Cigarettes: Cannabis | 15065.8 | (5585.85-24545.75) | 0.002 |  |  |  |
| Cigarettes: Analgesics: Cocaine | 3437.8 | (1250.87-5624.73) | 0.0022 |  |  |  |
| NHWhite.Cannabis | 2 | (0.73-3.27) | 0.0026 |  |  |  |
| Cigarettes: Analgesics | 13881.8 | (4913.11-22850.49) | 0.0026 |  |  |  |
| Cigarettes: Cannabis: Analgesics: Cocaine | 1167.2 | (409.64-1924.76) | 0.0027 |  |  |  |
| Cigarettes: Cannabis: Analgesics | 4717 | (1593.82-7840.18) | 0.0033 |  |  |  |
| Alcohol: Cocaine | 29956.9 | (10079.93-49833.87) | 0.0033 |  |  |  |
| Alcohol | 116488.8 | (35362.52-197615.08) | 0.0052 |  |  |  |
| Alcohol: Cannabis: Cocaine | 9890.4 | (2955.51-16825.29) | 0.0055 |  |  |  |
| Alcohol: Analgesics: Cocaine | 9054.8 | (2513.05-15596.55) | 0.007 |  |  |  |
| Alcohol: Cannabis | 38348.6 | (9877.05-66820.15) | 0.0087 |  |  |  |
| Alcohol: Analgesics | 35355.3 | (8616-62094.6) | 0.0099 |  |  |  |
| Alcohol: Cannabis: Analgesics: Cocaine | 2955.3 | (673.59-5237.01) | 0.0116 |  |  |  |
| Alcohol: Cannabis: Analgesics | 11491.4 | (2112.41-20870.39) | 0.0168 |  |  |  |
| NHAfric.Am | -0.2 | (-0.38--0.02) | 0.0361 |  |  |  |
| Cigarettes: Alcohol: Cannabis: Analgesics | -44585.5 | (-83013.59--6157.41) | 0.0236 |  |  |  |
| Cigarettes: Alcohol: Cannabis: Analgesics: Cocaine | -11208 | (-20538.09--1877.91) | 0.0191 |  |  |  |
| Cigarettes: Alcohol: Analgesics | -134314.4 | (-243691.57--24937.23) | 0.0166 |  |  |  |
| Cigarettes: Alcohol: Analgesics: Cocaine | -33719.9 | (-60413.22--7026.58) | 0.0137 |  |  |  |
| Cigarettes: Alcohol: Cannabis | -148642.8 | (-265262.04--32023.56) | 0.0129 |  |  |  |
| NHAIAN.Cannabis | -0.3 | (-0.5--0.1) | 0.0114 |  |  |  |
| Cigarettes: Alcohol: Cannabis: Cocaine | -37573.7 | (-65923.02--9224.38) | 0.0098 |  |  |  |
| Cigarettes: Alcohol | -442990.4 | (-774888.78--111092.02) | 0.0093 |  |  |  |
| NHAsian.Cannabis | -0.3 | (-0.54--0.06) | 0.0078 |  |  |  |
| Cigarettes: Alcohol: Cocaine | -111926.8 | (-193046.54--30807.06) | 0.0072 |  |  |  |
| Cannabis: Analgesics | -1185.9 | (-1950.77--421.03) | 0.0025 |  |  |  |
| Cannabis: Analgesics: Cocaine | -299.1 | (-485.1--113.1) | 0.0018 |  |  |  |
| Analgesics | -3563.9 | (-5763.76--1364.04) | 0.0016 |  |  |  |
| Cannabis | -3797.8 | (-6120.05--1475.55) | 0.0015 |  |  |  |
| Analgesics: Cocaine | -898 | (-1436.04--359.96) | 0.0012 |  |  |  |
| Cannabis: Cocaine | -962.9 | (-1528.16--397.64) | 0.0009 |  |  |  |
| Cocaine | -2876.2 | (-4511.86--1240.54) | 0.0006 |  |  |  |
| Hispanic.Cannabis | -0.8 | (-1.21--0.39) | 0.0002 |  |  |  |
| Median.Household.Income | -1.2 | (-1.51--0.89) | 0.0000 |  |  |  |
| NHAIAN | -0.2 | (-0.24--0.16) | 0.0000 |  |  |  |
