# Supplementary figures and images for: Impact of converging sociocultural and substance-related trends on US autism rates: combined geospatiotemporal and causal inferential analysis
Source: Eur Arch Psychiatry Clin Neurosci. 2022 Jul 2;273(3):699–717. doi: 10.1007/s00406-022-01446-0 (PMC10085966; doi:10.1007/s00406-022-01446-0)

# Major Drug, Ethnic and Income Paramaters, USA, 2000–2018

Data: DEA, US Census Bureau, NSDUH, SAMHSA

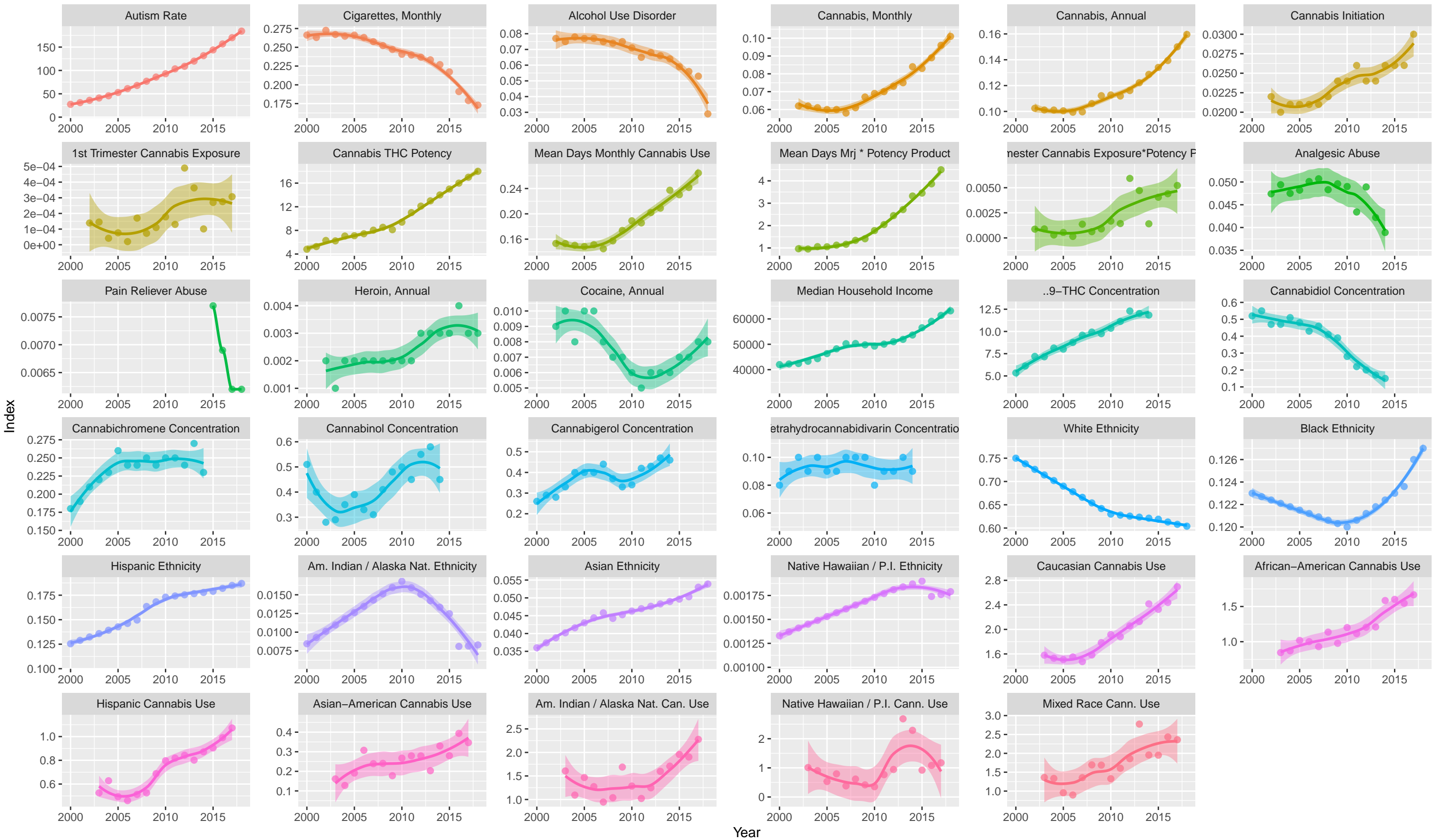

Supplement: Supplementary file 2 — Supplementary file2 (PDF 91 KB) [file 406_2022_1446_MOESM2_ESM.pdf]

# Cannabis Use by Frequency by Ethnicity

Data – NSDUH, SAMHSA, 2002–2017

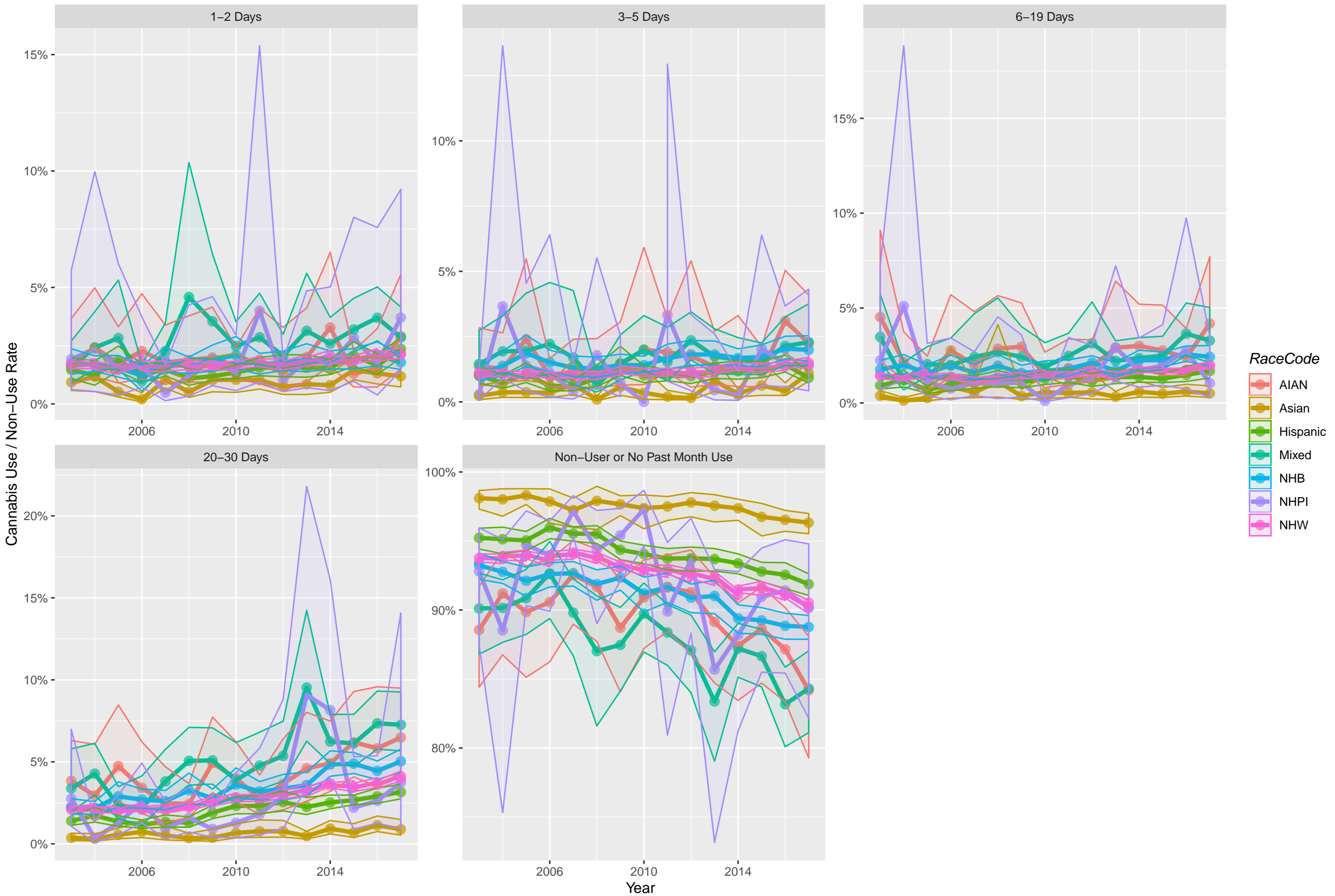

Supplement: Supplementary file 3 — Supplementary file3 (PDF 48 KB) [file 406_2022_1446_MOESM3_ESM.pdf]

### Sum of Race-Time Products by US State Index 2003–2011

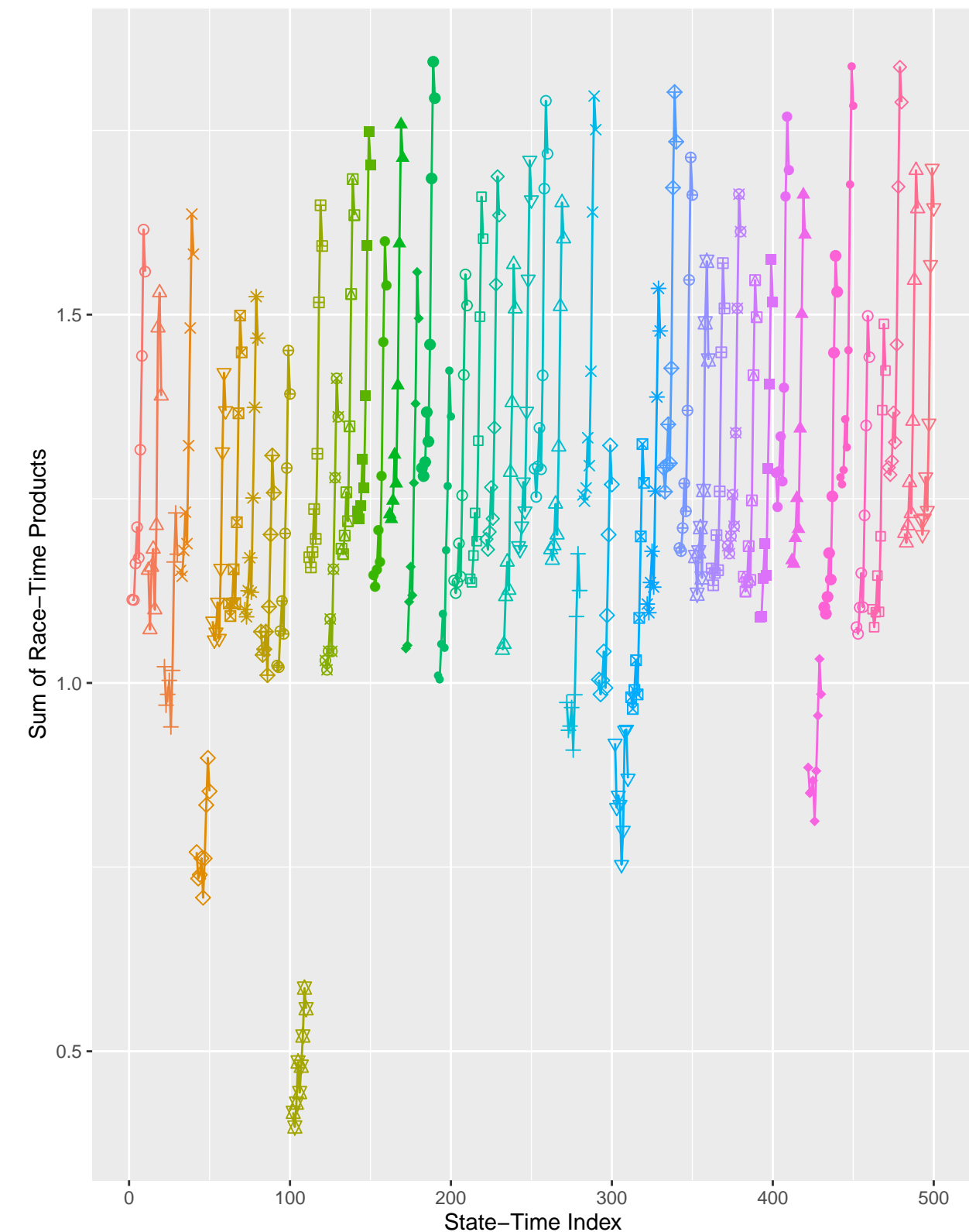

### Sum of Race-Time Products by US State Over Time 2003–2011

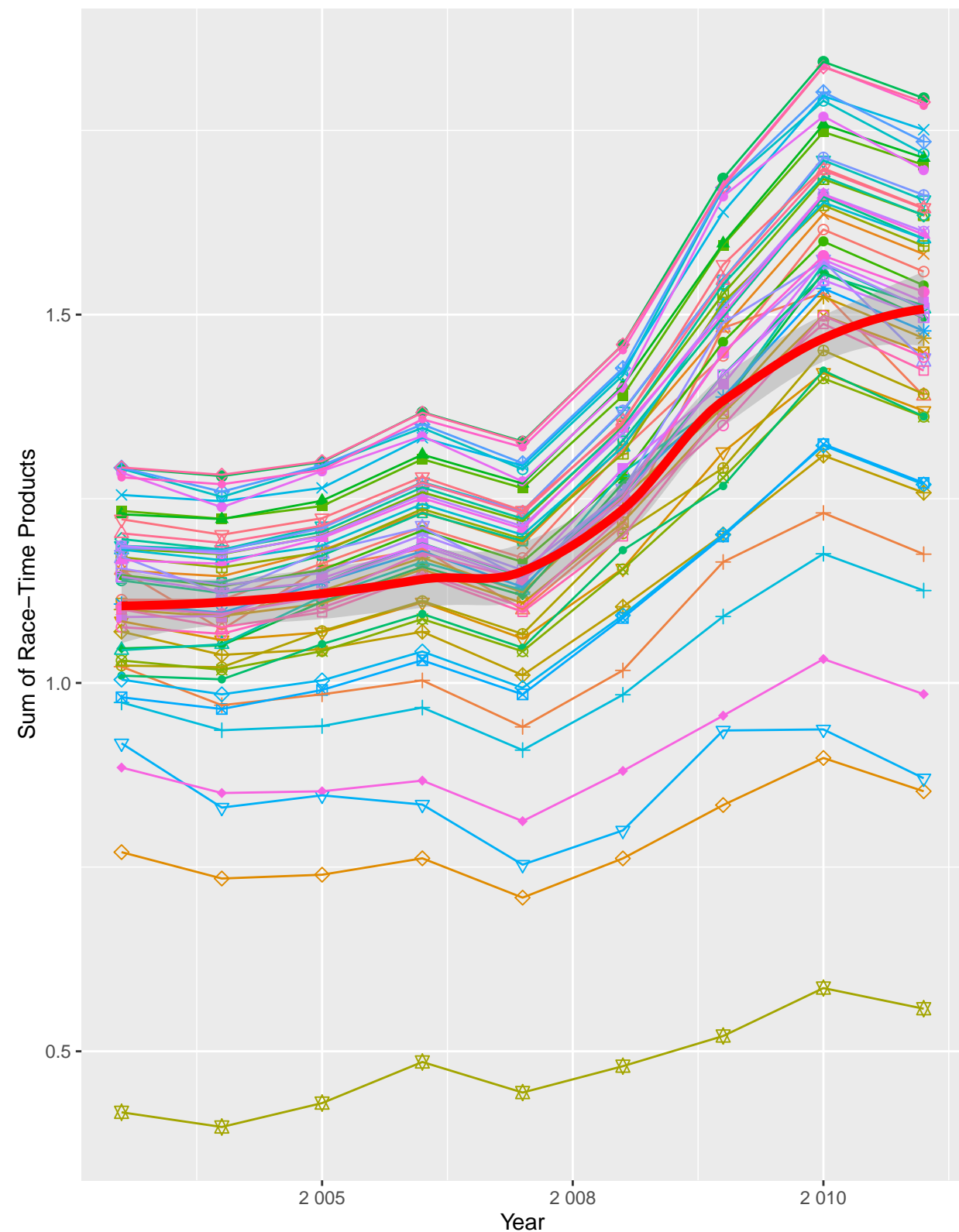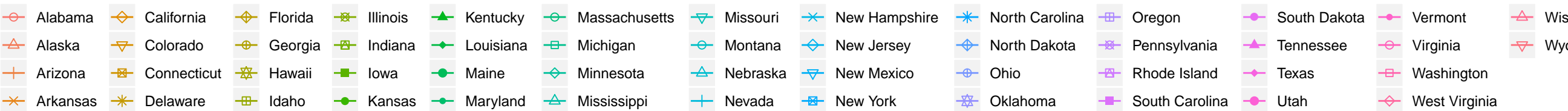

Supplement: Supplementary file 5 — Supplementary file5 (PDF 35 KB) [file 406_2022_1446_MOESM5_ESM.pdf]

Percent Daily / Near Daily Cannabis Use by Age Group,  
USA, NSDUH, SAMHSA

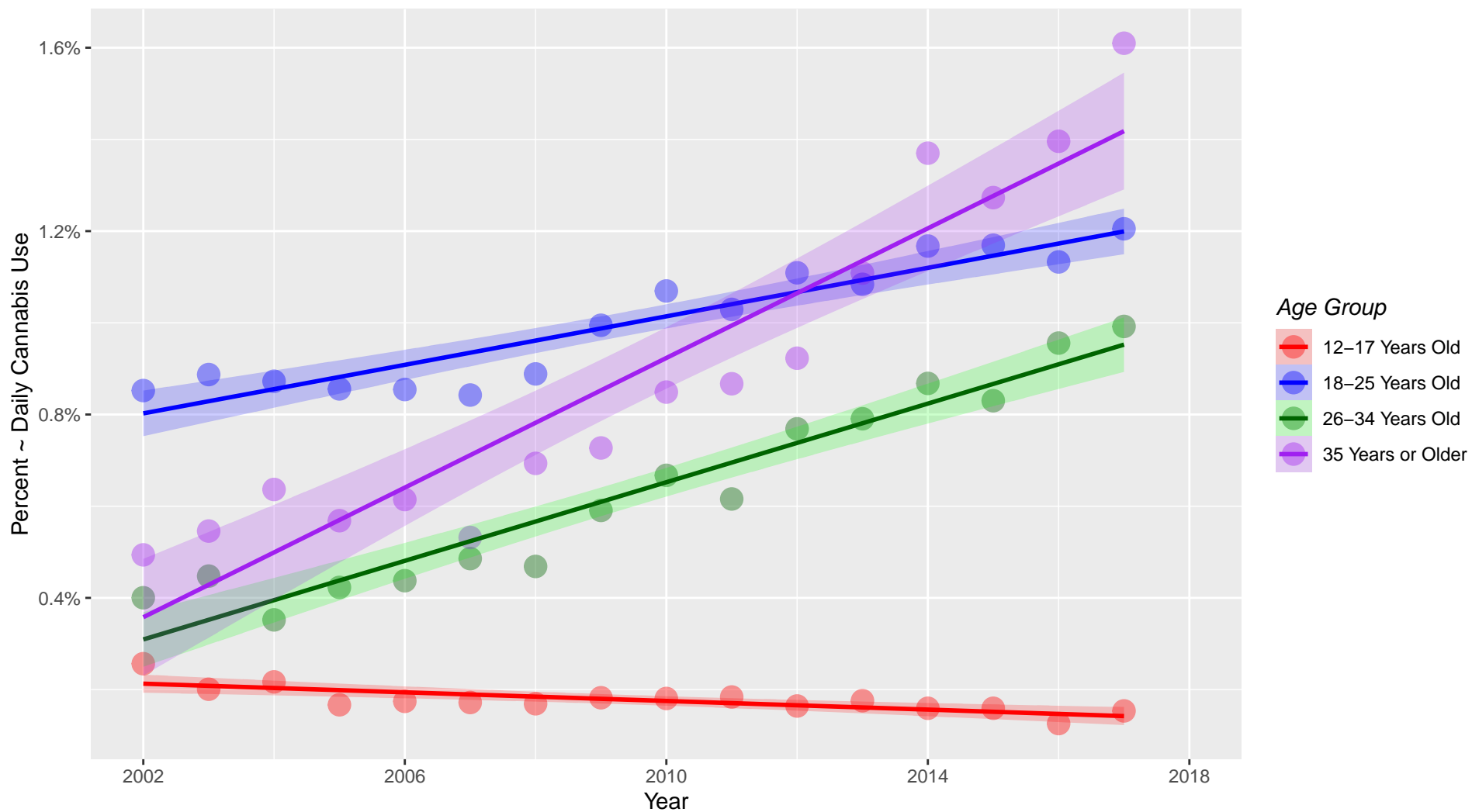

Supplement: Supplementary file 6 — Supplementary file6 (PDF 14 KB) [file 406_2022_1446_MOESM6_ESM.pdf]

# US Age of Maternal Child Bearing Over Time

## Data – CDC Wonder Births Registries

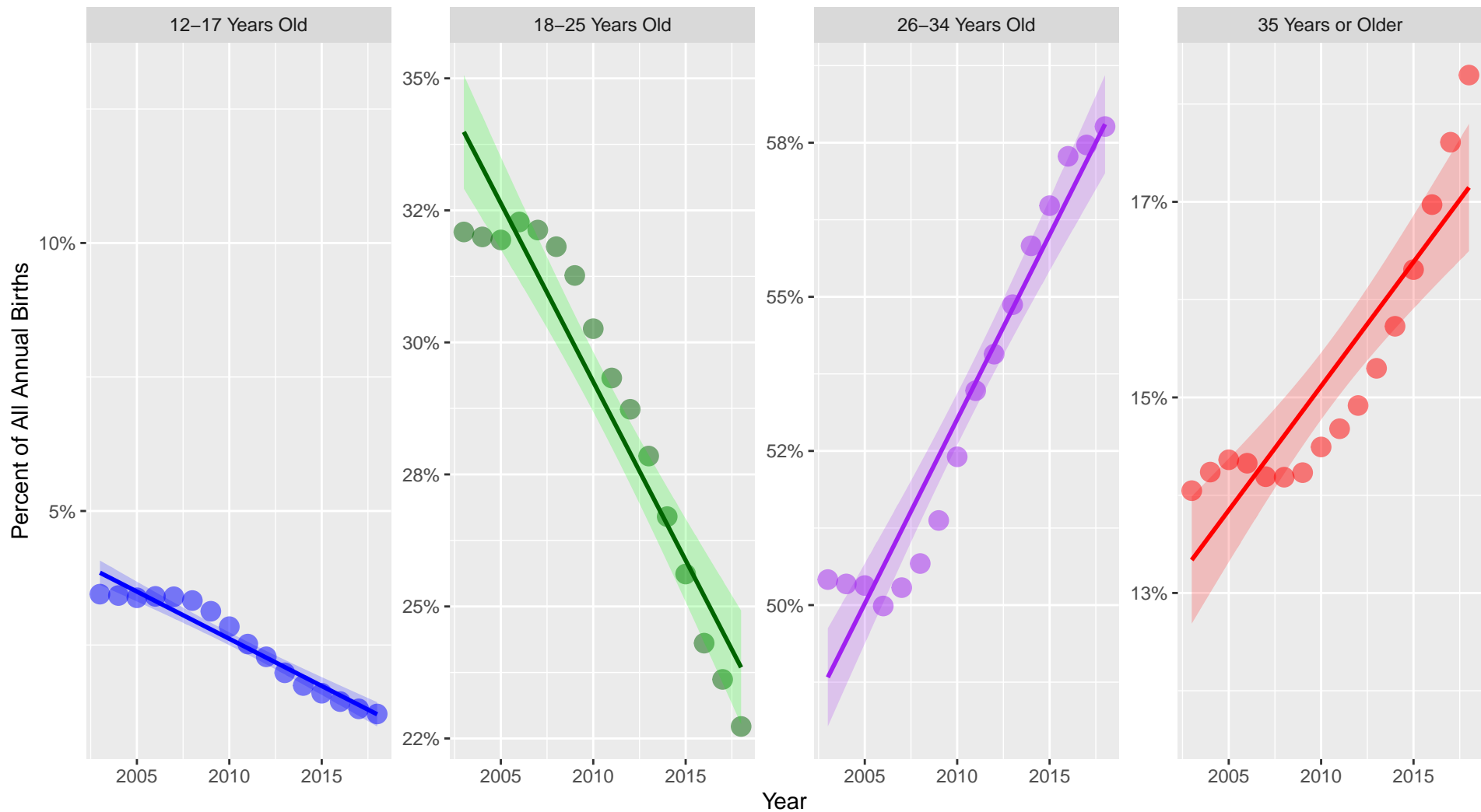

Supplement: Supplementary file 8 — Supplementary file8 (PDF 15 KB) [file 406_2022_1446_MOESM8_ESM.pdf]

# Autism Rate by Legal Status

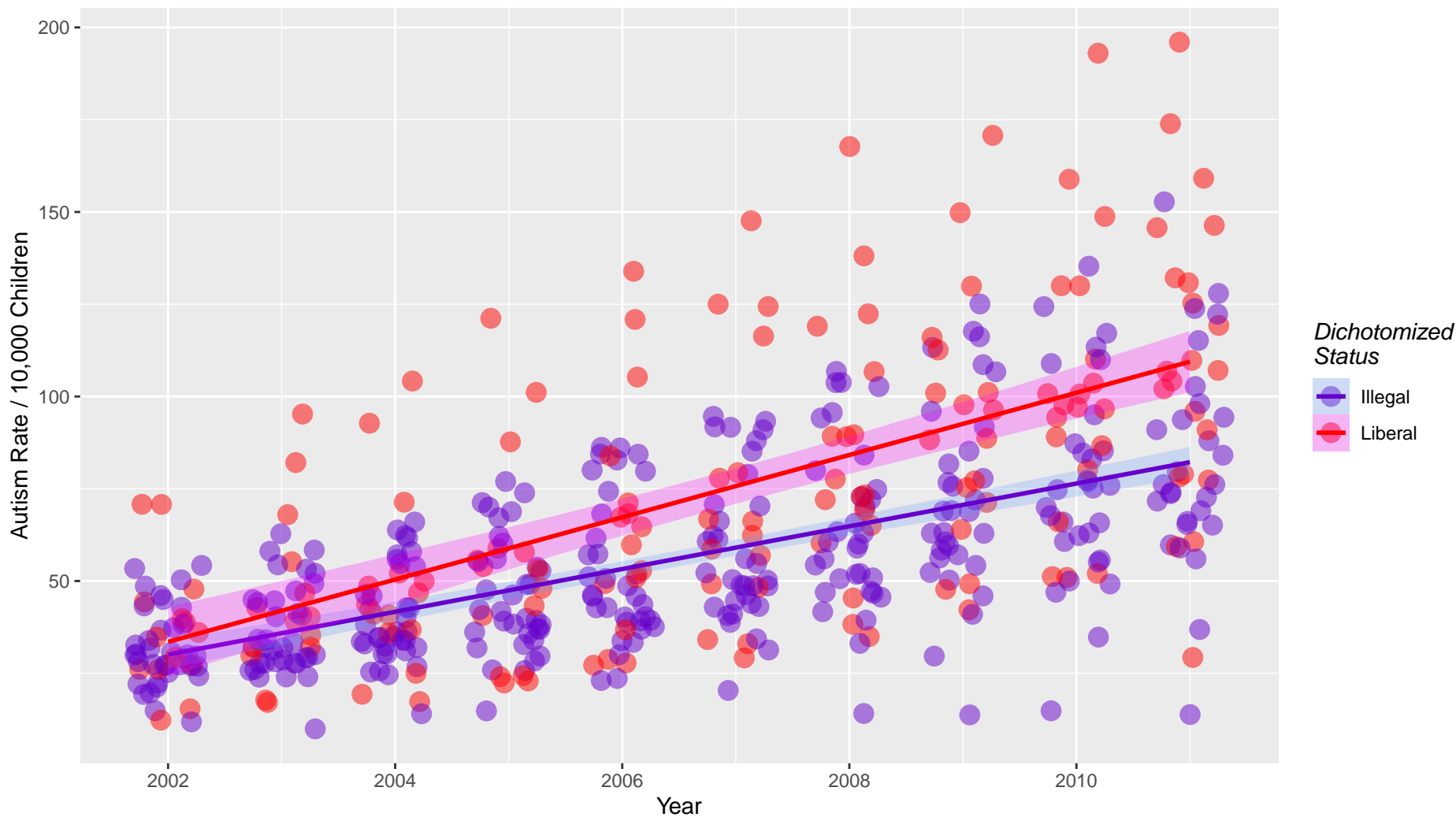

Supplement: Supplementary file 11 — Supplementary file11 (PDF 37 KB) [file 406_2022_1446_MOESM11_ESM.pdf]
