# Supplementary material for: Impact of converging sociocultural and substance-related trends on US autism rates: combined geospatiotemporal and causal inferential analysis
Source: Eur Arch Psychiatry Clin Neurosci. 2022 Jul 2;273(3):699–717. doi: 10.1007/s00406-022-01446-0 (PMC10085966; doi:10.1007/s00406-022-01446-0)
Supplement: Supplementary file 4 — Supplementary file4 (PDF 9 KB) [file 406_2022_1446_MOESM4_ESM.pdf]

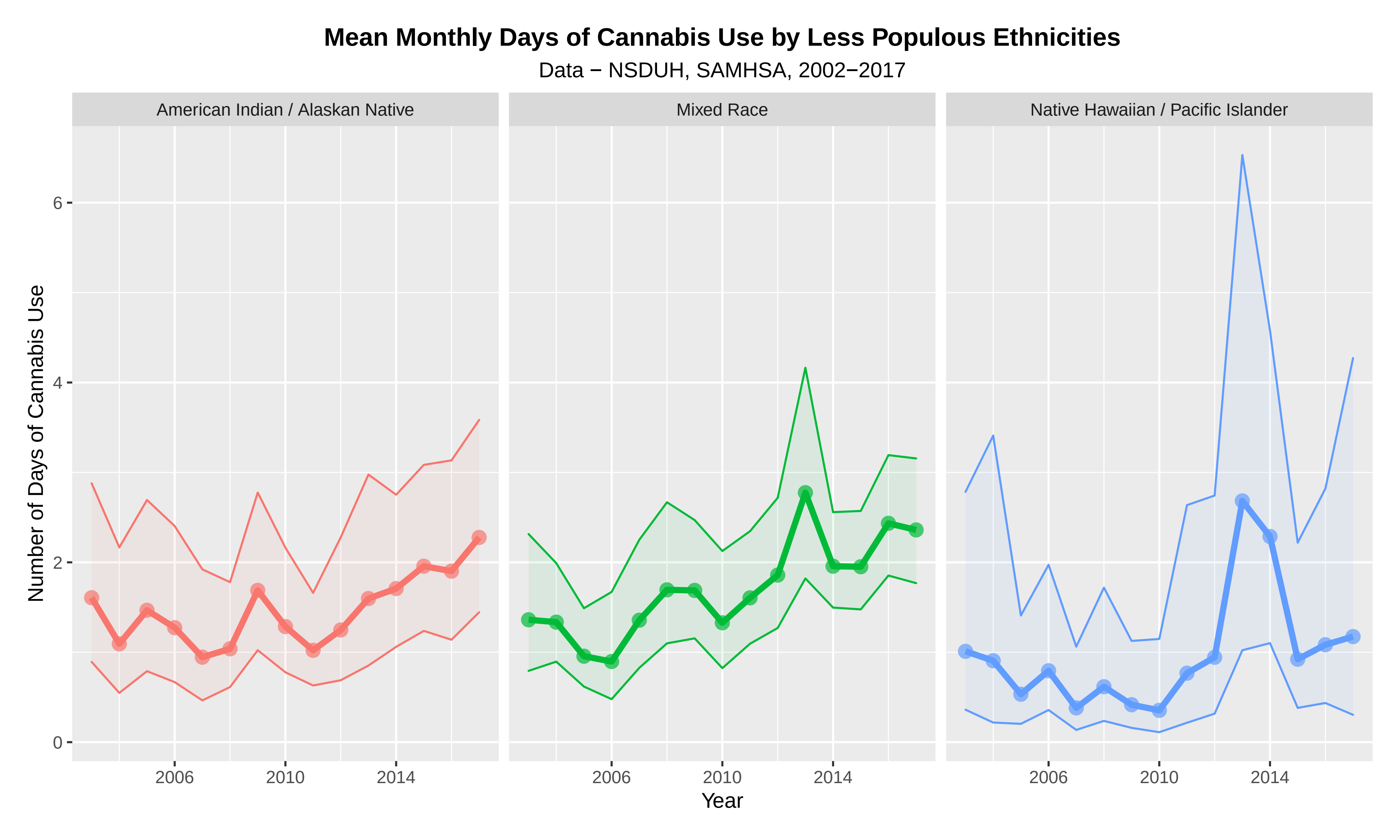
Mean Monthly Days of Cannabis Use by Less Populous Ethnicities
Data − NSDUH, SAMHSA, 2002−2017
American Indian / Alaskan Native
Mixed Race
Native Hawaiian / Pacific Islander
Number of Days of Cannabis Use
0
2
4
6
2006
2010
2014
Year
