# Supplementary material for: Impact of converging sociocultural and substance-related trends on US autism rates: combined geospatiotemporal and causal inferential analysis
Source: Eur Arch Psychiatry Clin Neurosci. 2022 Jul 2;273(3):699–717. doi: 10.1007/s00406-022-01446-0 (PMC10085966; doi:10.1007/s00406-022-01446-0)
Supplement: Supplementary file 7 — Supplementary file7 (PDF 27 KB) [file 406_2022_1446_MOESM7_ESM.pdf]

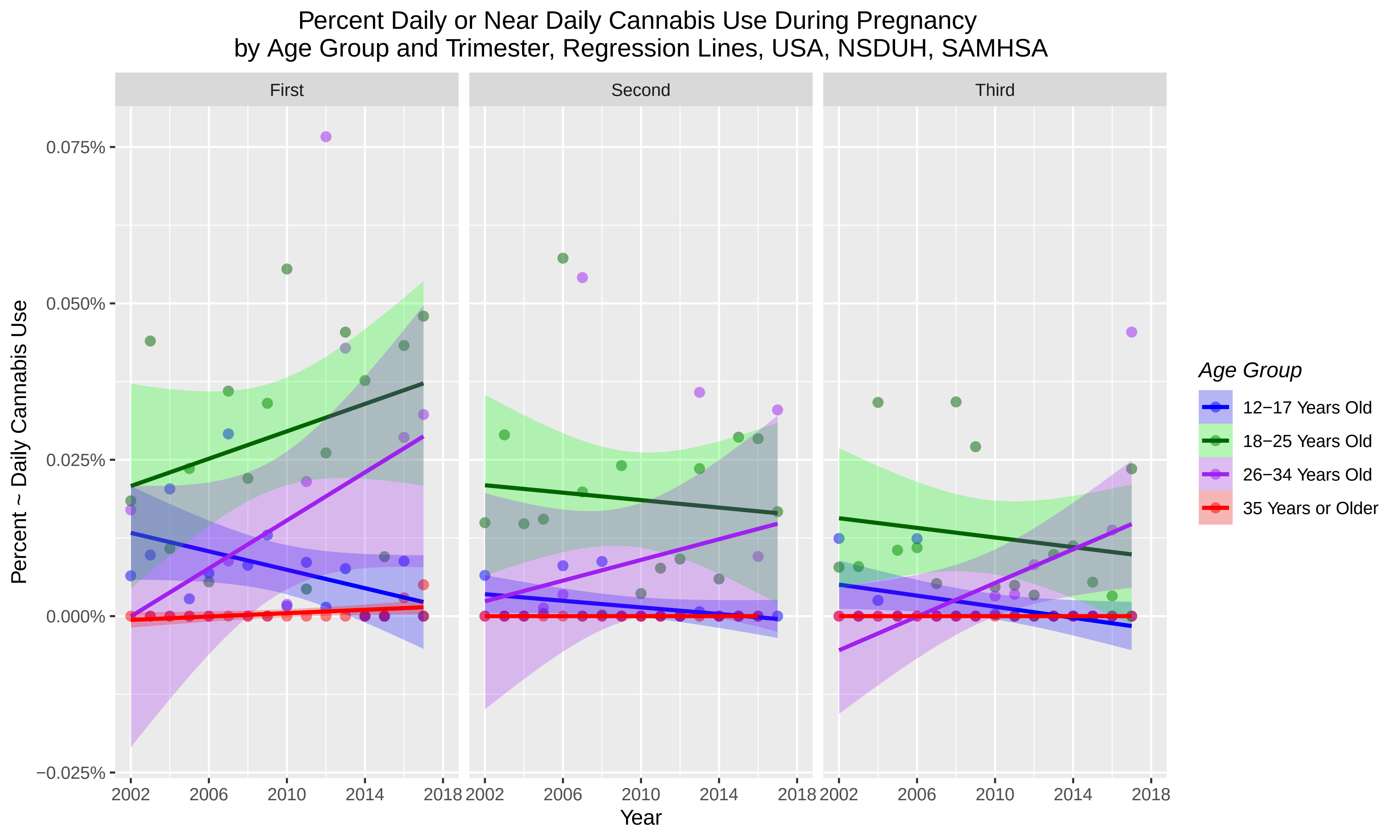

Percent Daily or Near Daily Cannabis Use During Pregnancy
by Age Group and Trimester, Regression Lines, USA, NSDUH, SAMHSA
First
Second
Third
0.075%
0.050%
0.025%
0.000%
−0.025%
Percent ~ Daily Cannabis Use
2002
2006
2010
2014
2018
Year
Age Group
12−17 Years Old
18−25 Years Old
26−34 Years Old
35 Years or Older
