# Supplementary material for: Impact of converging sociocultural and substance-related trends on US autism rates: combined geospatiotemporal and causal inferential analysis
Source: Eur Arch Psychiatry Clin Neurosci. 2022 Jul 2;273(3):699–717. doi: 10.1007/s00406-022-01446-0 (PMC10085966; doi:10.1007/s00406-022-01446-0)
Supplement: Supplementary file 9 — Supplementary file9 (PDF 2169 KB) [file 406_2022_1446_MOESM9_ESM.pdf]

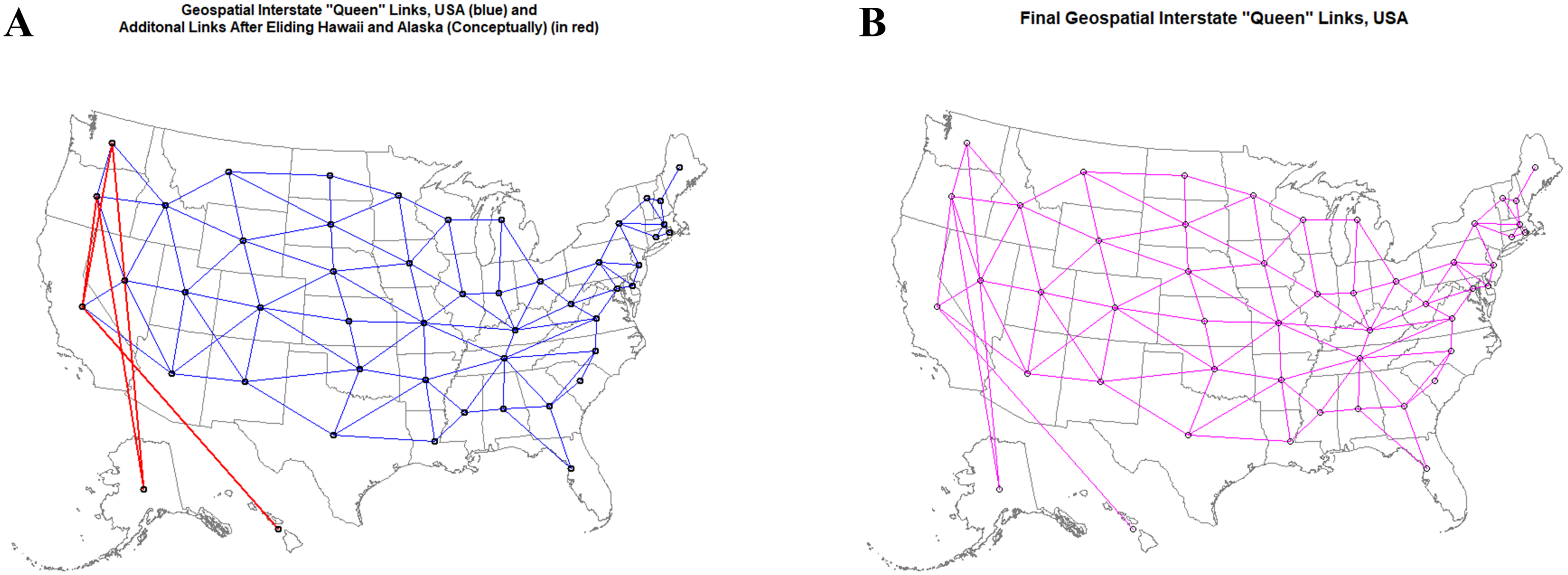
A
Geospatial Interstate "Queen" Links, USA (blue) and
Additonal Links After Eliding Hawaii and Alaska (Conceptually) (in red)
B
Final Geospatial Interstate "Queen" Links, USA
