# Supplementary material for: Impact of converging sociocultural and substance-related trends on US autism rates: combined geospatiotemporal and causal inferential analysis
Source: Eur Arch Psychiatry Clin Neurosci. 2022 Jul 2;273(3):699–717. doi: 10.1007/s00406-022-01446-0 (PMC10085966; doi:10.1007/s00406-022-01446-0)
Supplement: Supplementary file 10 — Supplementary file10 (PDF 5461 KB) [file 406_2022_1446_MOESM10_ESM.pdf]

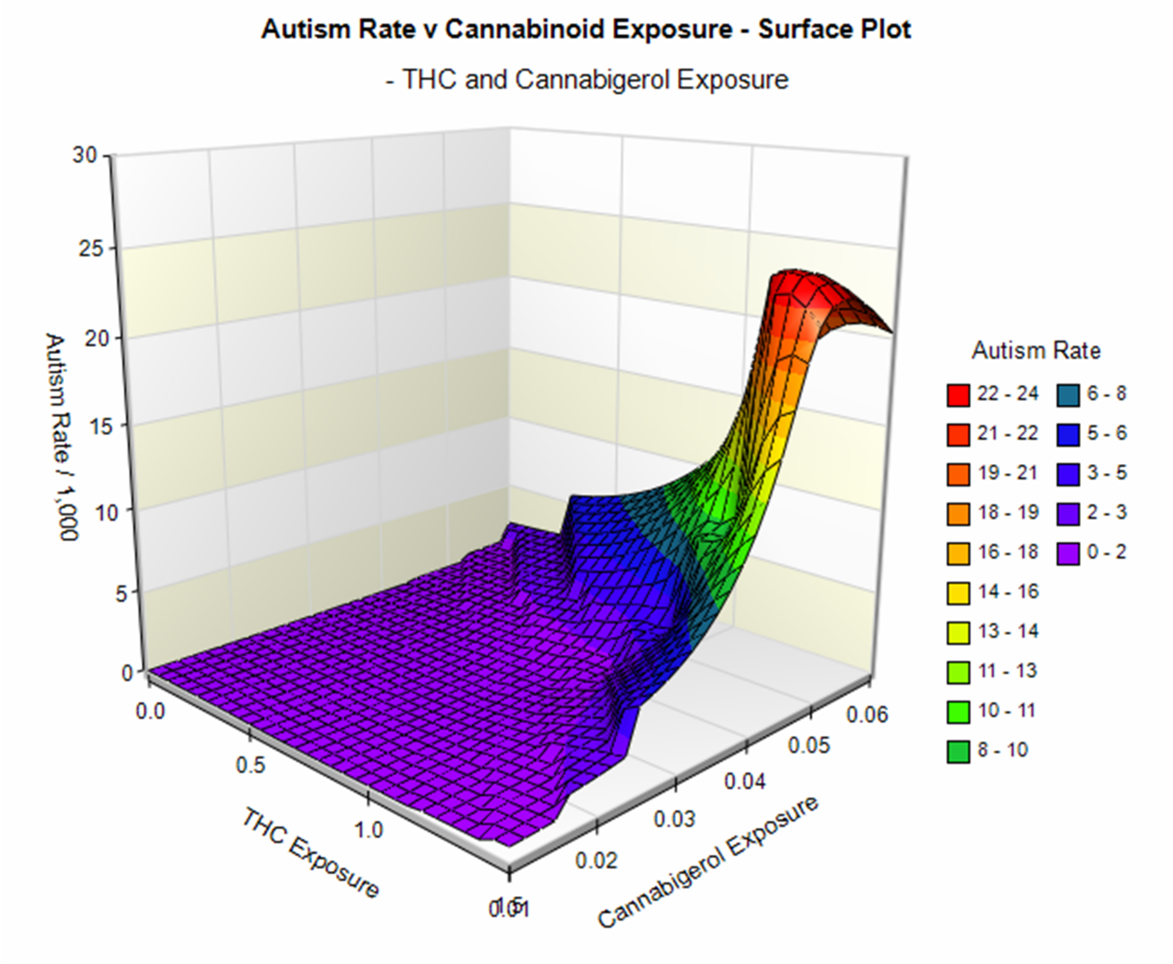

Autism Rate v Cannabinoid Exposure - Surface Plot
- THC and Cannabigerol Exposure
Autism Rate / 1,000
30
25
20
15
10
5
0
0.0
0.5
1.0
THC Exposure
0.02
0.03
0.04
0.05
0.06
Cannabigerol Exposure
Autism Rate
22 - 24
21 - 22
19 - 21
18 - 19
16 - 18
14 - 16
13 - 14
11 - 13
10 - 11
8 - 10
6 - 8
5 - 6
3 - 5
2 - 3
0 - 2

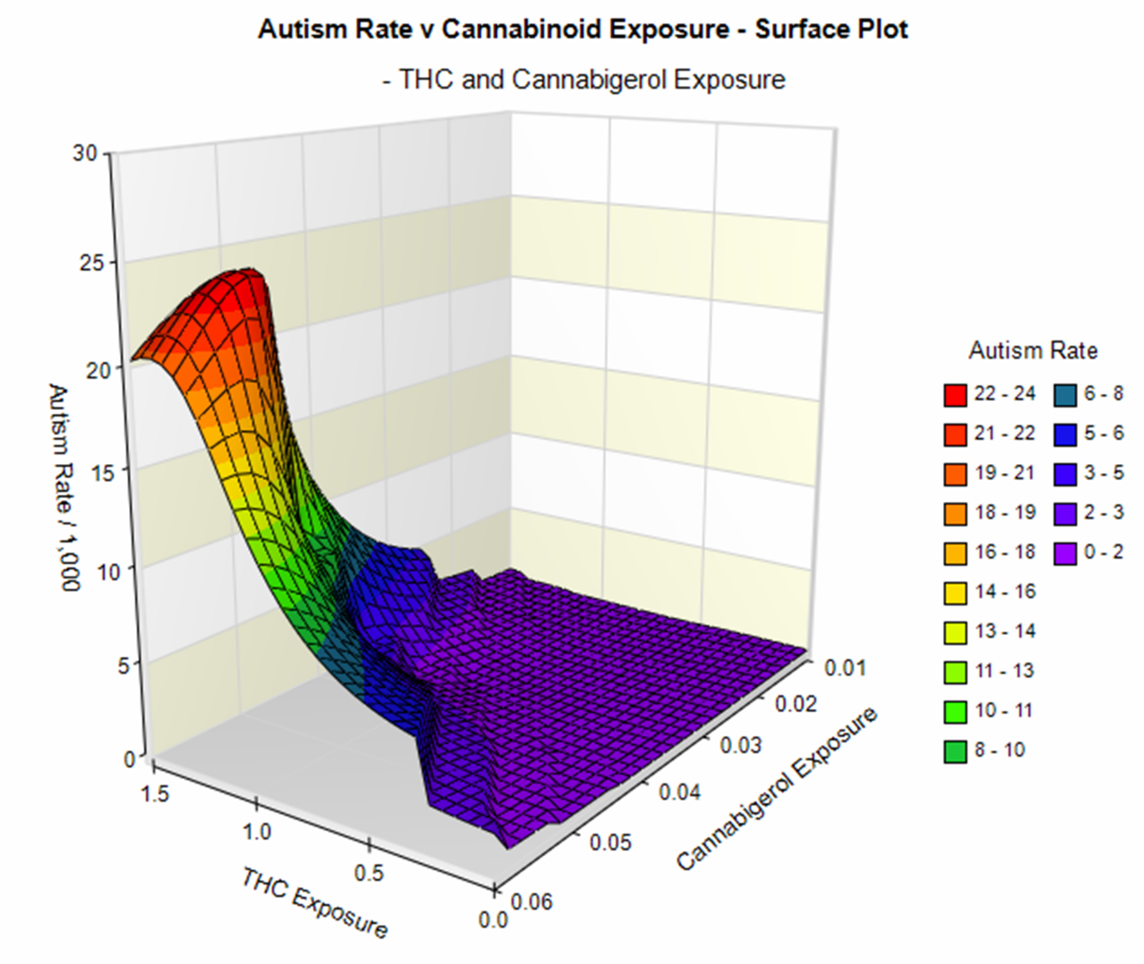

Autism Rate v Cannabinoid Exposure - Surface Plot
- THC and Cannabigerol Exposure
Autism Rate / 1,000
30
25
20
15
10
5
0
1.5
1.0
0.5
0.0
THC Exposure
0.01
0.02
0.03
0.04
0.05
0.06
Cannabigerol Exposure
Autism Rate
22 - 24
21 - 22
19 - 21
18 - 19
16 - 18
14 - 16
13 - 14
11 - 13
10 - 11
8 - 10
6 - 8
5 - 6
3 - 5
2 - 3
0 - 2
